# Investigation of the Autoregulator-Receptor System in the Pristinamycin Producer *Streptomyces pristinaespiralis*

**DOI:** 10.3389/fmicb.2020.580990

**Published:** 2020-09-30

**Authors:** Franziska Handel, Andreas Kulik, Yvonne Mast

**Affiliations:** ^1^Department of Microbiology/Biotechnology, Faculty of Science, Interfaculty Institute of Microbiology and Infection Medicine, University of Tübingen, Tübingen, Germany; ^2^German Center for Infection Research (DZIF), Partner Site Tübingen, Tübingen, Germany; ^3^Department Bioresources for Bioeconomy and Health Research, Leibniz Institute DSMZ – German Collection of Microorganisms and Cell Cultures, Braunschweig, Germany; ^4^Department of Microbiology, Technical University Braunschweig, Braunschweig, Germany

**Keywords:** *Streptomyces*, antibiotics, transcriptional regulation, signal molecules, γ-butyrolactone

## Abstract

Pristinamycin biosynthesis in *Streptomyces pristinaespiralis* is governed by a complex hierarchical signaling cascade involving seven different transcriptional regulators (SpbR, PapR1, PapR2, PapR3, PapR4, PapR5, and PapR6). The signaling cascade is triggered by γ-butyrolactone (GBL)-like effector molecules, whereby the chemical structure of the effector, as well as its biosynthetic origin is unknown so far. Three of the pristinamycin transcriptional regulators (SpbR, PapR3, and PapR5) belong to the type of γ-butyrolactone receptor (GBLR). GBLRs are known to either act as “real” GBLRs, which bind GBLs as ligands or as “pseudo” GBLRs binding antibiotics or intermediates thereof as effector molecules. In this study, we performed electromobility shift assays (EMSAs) with SpbR, PapR3, and PapR5, respectively, in the presence of potential ligand samples. Thereby we could show that all three GBLRs bind synthetic 1,4-butyrolactone but not pristinamycin as ligand, suggesting that SpbR, PapR3, and PapR5 act as “real” GBLRs in *S. pristinaespiralis*. Furthermore, we identified a cytochrome P450 monooxygenase encoding gene *snbU* as potential biosynthesis gene for the GBLR-interacting ligand. Inactivation of *snbU* resulted in an increased pristinamycin production, which indicated that SnbU has a regulatory influence on pristinamycin production. EMSAs with culture extract samples from the *snbU* mutant did not influence the target binding ability of SpbR, PapR3, and PapR5 anymore, in contrast to culture supernatant samples from the *S. pristinaespiralis* wild-type or the pristinamycin deficient mutant *papR2::apra*, which demonstrates that SnbU is involved in the synthesis of the GBLR-interacting ligand.

## Introduction

Bacteria of the genus *Streptomyces* are filamentous, gram-positive soil bacteria, which are characterized by a complex morphological life-cycle and their ability to produce a wide variety of secondary metabolites, including the majority of all known antibiotics (Tanaka and Omura, 1990; Liu et al., 2013). Antibiotic production in these organisms is usually subject to control through complex regulatory networks, which respond to various environmental and physiological factors (McCormick and Flärdh, 2012). One common important initiation principle that triggers antibiotic production in streptomycetes is governed by a quorum sensing-like system employing γ-butyrolactones (GBLs) as signaling molecules. GBLs are small, diffusible molecules referred to as microbial hormones, which are produced by at least 60% of all *Streptomyces* species, where they induce antibiotic production in nanomolar concentrations (Takano et al., 2000; Liu et al., 2013; Niu et al., 2016). Depending on the type of GBL, the molecules are synthesized by the action of only one, or multiple enzymes: e.g., AfsA synthesizes the A-factor GBL in the streptomycin producer *Streptomyces griseus* (Ando et al., 1997) and ScbA is responsible for the synthesis of the SCBs in the actinorhodin (ACT)/undecylprodigiosin (RED) producer *Streptomyces coelicolor* (Hsiao et al., 2007), whereby three enzymes (BarX, BarS1, and BarS2) have been shown to be involved in virginiae butanolide (VB) biosynthesis in the virginiamycin producer *Streptomyces virginiae* (Shikura et al., 2002; Lee et al., 2008, 2010). On the molecular level, GBLs bind to their cognate GBL receptor (GBLR), which resemble TetR-like family regulators that usually act as transcriptional repressors by binding to specific sequence motifs (ARE motifs) within the promoter regions of their target genes. TetR-like regulators bind to the DNA regions as “Ω” shaped dimers (Ramos et al., 2005). They consist of a helix-turn-helix (HTH) DNA-binding motif in the N-terminal domain, and a ligand-binding pocket in the C-terminal domain (Yu et al., 2010). Upon GBL binding, the GBLR undergoes a conformational change, whereby it is released from the promoter region(s), which in turn allows transcription of the previously repressed gene(s) (Ramos et al., 2005). Well-known examples for GBLR regulators in streptomycetes are ArpA from *S. griseus*, which binds the A-factor as a ligand or ScbR from *S. coelicolor* responding to the SCB1 molecule (Takano et al., 2008). GBLR target genes often involve additional transcriptional regulatory genes, which ends up in quite complex hierarchical signaling cascades employing pleiotropic but also pathway-specific transcriptional regulators (Bibb, 2005).

Besides the above described “real” GBLR regulators also homologous regulator-types are present in streptomycetes called “pseudo” GBLRs (pGBLRs), which instead of GBLs bind antibiotics or antibiotic intermediates as ligands (Xu et al., 2010). The first described and best characterized pGBLRs are ScbR2 from *S. coelicolor* and JadR2 from *Streptomyces venezuelae*, which both bind endogenous antibiotics from these bacteria as ligands, as there are: ACT and RED in terms of ScbR2, and jadomycin and chloramphenicol in terms of JadR2 (Xu et al., 2010; Table 1). Interestingly, these antibiotics bind to their associated receptors even if they have a complete distinct chemical structure. The pGBLRs act in concert with the genuine GBLRs in these strains, which are ScbR in *S. coelicolor*, with the associated SCB ligands (Xu et al., 2010) and JadR1/3 in *S. venezuelae*, with the cognate SVB ligands (Zou et al., 2014). Together they control the transcriptional regulation of the respective antibiotic biosynthesis. Further examples of such GBLR/pGBLR combinations have been suggested for the virginiamycin producer *S. virginiae* (Table 1). Here, BarA has been shown to function as the GBLR, which binds VBs as ligands, whereas BarB is the proposed pGBLR (Matsuno et al., 2004). Accordingly, SagR is the suggested GBLR in the auricin producer *Streptomyces aureofaciens* and Aur1R the pGBLR (Mingyar et al., 2015). In *Streptomyces lavendulae*, which produces the blue pigment indigoidine, cycloserine and nucleoside antibiotics, FarA is the GBLR that binds the IM-2 effector molecule, whereas FarR2 is the suggested pGBLR (Kurniawan et al., 2016). Based on phylogenetic comparisons between different GBLR-like regulators from streptomycetes, BulR1 from the tacrolimus (FK506) producer *Streptomyces tsukubaensis* is the proposed GBLR and BulR2 the pGBLR (Salehi-Najafabadi et al., 2014). This assignment has also been done due to the different p*I* values of both regulator types (BulR1: p*I* 5.51; BulR2: p*I* 10.15). p*I* differences have previously been suggested to serve as a criterion to classify GBLRs and pGBLRs, whereby GBLRs tend to have a more acidic p*I* value and pGBLRs a more basic one (Lee et al., 2008; Kitani et al., 2008). This was also observed for the GBLR/pGBLR combination BarA/BarB of *S. virginiae* (Kinoshita et al., 1997; Kawachi et al., 2000) or FarA/FarR2 of *S. lavendulae* (Kawachi et al., 2000; Kitani et al., 2008). In the various antibiotic producers GBLRs and pGBLRs act in concert and together fine-tune secondary metabolite production, which can end up in complex feed-forward/feedback loops (Xu and Yang, 2019).

**TABLE 1:** GBLRs and pGBLRs from different antibiotic-producing streptomycetes.

Strain	GBLR	pGBLR	Antibiotic	References
*S. acidiscabies*	SabR	SabS	Thaxtomin	Kitani et al., 2008
S. ambofaciens*	AlpZ	AlpW	Alpomycin	Bunet et al., 2011
*S. ansochromogenes*	SabR1	SabR2	Nikkomycin	Wang et al., 2018
S. aureofaciens*	SagR	Aur1R	Auricin	Wang et al., 2011
S. avermitilis*	AvaR1, AvaR3	AvaR2	Avermectin	Zhu et al., 2016
S. coelicolor*	ScbR	ScbR2, CprB	Actinorhodin, Undecylprodigiosin	Xu et al., 2010
*S. fradiae*	TylP	TylQ	Tylosin	Bignell et al., 2007
*S. griseus*	ArpA	SGR6382	Streptomycin	Horinouchi, 2007
S. lavendulae*	FarA	FarR2	Indigoidine, Cycloserine, Nucleoside antibiotics	Kurniawan et al., 2016
*S. rochei*	SrrA	SrrB	Lankamycin	Arakawa et al., 2012
S. scabies*	SscR	SscF	Thaxtomin	Kitani et al., 2008
S. tsukubaensis*	BulR1	BulR2	Tacrolimus	Salehi-Najafabadi et al., 2014
S. venezuelae*	JadR1/3	JadR2	Jadomycin	Zou et al., 2014
*S. virginiae*	BarA	BarB	Virginiamycin	Nishida et al., 2007

*Experimentally verified pGBLR function is marked with an asterisk.*

Such a complex regulatory system is also present in the pristinamycin producer *Streptomyces pristinaespiralis* (Mast et al., 2011, 2015). Pristinamycin is a streptogramin antibiotic consisting of the peptide antibiotic pristinamycin I and the polyketide antibiotic pristinamycin II (Mast and Wohlleben, 2014). Regulation of pristinamycin biosynthesis is governed by a GBL quorum sensing-like system (Mast et al., 2015). So far, the GBLR-interacting ligand molecule(s) as well as its encoding biosynthetic gene(s) have not been identified. In a previous study, endogenous factors have been isolated from *S. pristinaespiralis* cultures, which showed an inducing effect on pristinamycin production (Paquet et al., 1992). Furthermore, the addition of the A-factor, as well as several commercial GBLs resulted in an induction of pristinamycin production in *S. pristinaespiralis*, whereby the A-factor showed the strongest inducing effect (Paquet et al., 1992). Thus, it has been suggested that the pristinamycin-specific effector may resemble a A-factor-like GBL (Paquet et al., 1992). The reported cognate GBLR in *S. pristinaespiralis* is SpbR (*S. pristinaespiralis* butyrolactone-responsive transcriptional repressor) (Folcher et al., 2001), which acts as the pleiotropic regulator of the pristinamycin signaling cascade (Mast et al., 2015).

**FIGURE 1:**
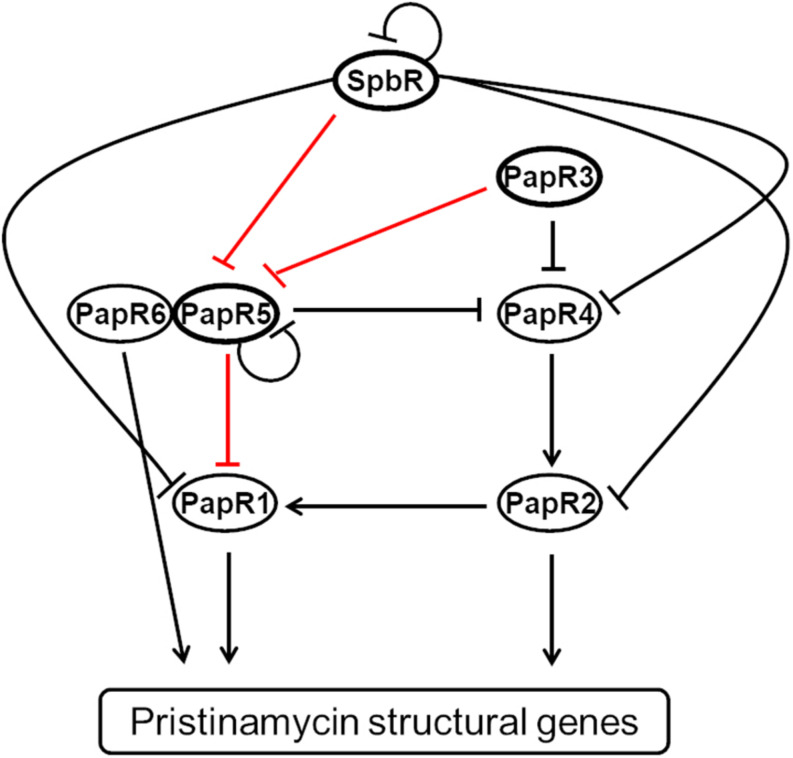
Model of the regulation of the pristinamycin regulatory signaling cascade of *S. pristinaespiralis* according to Mast et al. (2015). Binding of pristinamycin regulators is indicated by arrows (activating effect) and perpendicular lines (repressive effect). The GBLR-like regulators SpbR, PapR3, and PapR5 used in this study are highlighted by bold lined ellipses. The tested promoter interactions are indicated as red lines.

The signaling cascade comprises six additional pristinamycin-specific transcriptional regulators, which are encoded by the genes *papR1*, *papR2*, *papR3*, *papR4*, *papR5*, and *papR6* (Mast et al., 2015; Figure 1). *papR1*, *2, 4*, and *6* code for transcriptional activators, whereby the encoded PapR1, 2, and 4 proteins belong to the SARP-type family of transcriptional regulators and PapR6 represents a response regulator. *papR3* and *papR5* encode the TetR-like regulators PapR3 and PapR5, which act as transcriptional repressors of pristinamycin biosynthesis (Mast et al., 2015). The regulatory function of the pristinamycin regulators has been shown by mutagenesis and overexpression experiments, as well as RT-PCR and EMSA studies (Mast et al., 2015). Regarding the pristinamycin-specific TetR-like regulators, inactivation of *papR3* and *papR5* resulted in a up to 150 and 300% increase of pristinamycin production, respectively (Mast et al., 2015). The *papR5* mutant additionally showed a morphological defect and lacked the formation of aerial mycelium and spores, which illustrated the pleiotropic regulatory function of the encoded PapR5 regulator (Mast et al., 2015).

On the molecular level, SpbR binds to its own promoter region, as well as to the promoter regions of nearly all *papR* regulators, except for *papR3* and *papR6*. PapR3 binds to the promoter regions of *papR5* and *papR4*, whereas PapR5 binds to its own promoter region and to the promoter region of the SARP gene *papR1. papR1* transcription is also under control of the SARP-type regulator PapR2, which is the essential activator of pristinamycin biosynthesis that binds to promoter regions of the pristinamycin I and II structural genes (Mast et al., 2015; Figure 1). It has been found that all GBLR-like regulators SpbR, PapR3, and PapR5 have a conserved Trp residue at position 191, which usually serves as a binding site for GBLs in homologous known GBL receptors (Mast, 2008), suggesting that all three regulators may act as GBLRs. According to the classification of Salehi-Najafabadi et al. (2014) PapR3 with a basic p*I* of 9.92 would be a pGBLR, whereas SpbR and PapR5 with an acidic p*I* of 5.72 and 6.08, respectively, should be “real” GBLRs (Mast et al., 2015). However, this association is in conflict with a phylogenetic study of various TetR-like regulators from different streptomycetes, according to which PapR5 was assigned to the pGBLRs and SpbR and PapR3 to GBLRs (Cuthbertson and Nodwell, 2013). Thus, experimental research is necessary to ascertain if SpbR, PapR3, and PapR5 act as “real” or “pseudo” GBLRs in *S. pristinaespiralis.*

Here, we report on EMSA studies with SpbR, PapR3, and PapR5 in the presence of various potential ligand samples, such as commercial GBLs, pristinamycin or *S. pristinaespiralis* culture extract samples and thereby demonstrate that all three GBLR-like regulators act as real GBLRs in *S. pristinaespiralis.* Furthermore, we report on the identification and functional analysis of the potential GBLR-interacting ligand biosynthesis gene *snbU.*

## Results

### SpbR, PapR3, and PapR5 Are GBLR-Like Regulators With DNA Binding Ability

In a previous study it has been shown that SpbR, PapR3, and PapR5 from *S. pristinaespiralis* are TetR-like regulators that bind to promoter regions of pristinamycin regulatory genes (Mast et al., 2015; Figure 1). In order to perform ligand-dependent regulator-DNA interaction analysis, the experimental conditions had to be predefined in order to allow for proper regulator-DNA interaction studies. For these analyses EMSAs were performed with the GBLR-type regulators SpbR, PapR3, and PapR5 together with representative cognate promoter regions. P*papR5* was used as a representative promoter region for SpbR and PapR3, whereas P*papR1* served as test promoter fragment for PapR5 (Supplementary Figure S1). For EMSA studies, cell lysates from the respective *Streptomyces lividans* overexpression strains *SLspbR-OE, SLpapR3-OE*, and *SLpapR5-OE* expressing the GBLRs as His-tagged proteins were used together with the 276 and 291 bp Cy5-labeled promoter regions P*papR1* and P*papR5*, respectively, (Supplementary Table S1 and Supplementary Figure S1, band 3). The respective promoter fragment without the addition of lysate served as internal reference (Supplementary Figure S1, band 1). As a control, the shifts were carried out with cell lysates from *S. lividans* harboring the empty pGM190 vector (*SLpGM190*) (Supplementary Table S1 and Supplementary Figure S1, band 2). Shift specificity was verified by the addition of increasing concentrations of competitive unlabeled target DNA, which successively out-competed the specific binding interaction (Supplementary Figure S1, band 4–6), as well as competitive unspecific DNA, which did not influence binding interaction (Supplementary Figure S1, band 7). EMSA assays revealed that SpbR and PapR3 cell lysate samples lead to a specific shift of the P*papR5* promoter fragment, whereas the PapR5 cell lysate samples led a shifted P*papR1* band (Supplementary Figure S1, band 3). Thus, EMSAs verified that SpbR and PapR3 specifically bound to the promoter region of *papR5*, and PapR5 specifically bound to the promoter region of *papR1.*

#### DNA Binding Activity of Pristinamycin-Specific GBLR-Like Regulators Is Affected by Synthetic 1,4-Butyrolactone

**FIGURE 2:**
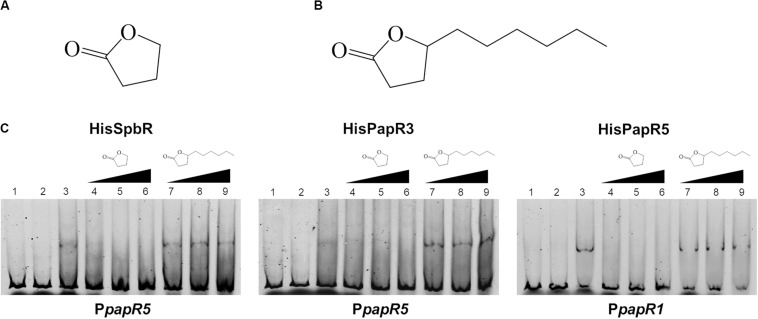
Chemical structure of BL **(A)** and GDL **(B)**. EMSAs **(C)** performed in 5% acrylamide gel with cell lysate samples from *SLspbR-OE* (left), *SLpapR3-OE* (middle) with P*papR5* and *SLpapR5-OE* (right) with P*papR1* in the presence of synthetic GBLs. 1, promotor DNA; 2, promotor DNA + *SLpGM190* lysate; 3, promotor DNA + cell lysate from GBLR overexpression sample; 4–6, promotor DNA + cell lysate from GBLR overexpression sample + increasing concentration (1, 2, 4 μl, respectively) of BL; 7–9, promotor DNA + cell lysate from GBLR overexpression sample + increasing concentration (1, 2, 4 μl, respectively) of GDL.

In a previous study from Paquet et al. (1992) it has been shown that pristinamycin biosynthesis is induced by the addition of different commercial GBLs. In order to ascertain if the pristinamycin-specific GBLR-like regulators function as receptors for GBLs as ligands, EMSAs were performed with SpbR, PapR3, and PapR5 in the presence of selected commercially available GBLs. As representative GBLs, synthetic 1,4-butyrolactone (BL) (C_4_H_6_O_2_, Figure 2A) and γ-decanolactone (GDL) (C_10_H_18_O_2_, Figure 2B) were tested. BL was used as a synthetic A-factor analog. A-factor and GDL both induced pristinamycin production in *S. pristinaespiralis* at minimal concentrations (0.001 and 0.25 g/ml, respectively) before (Paquet et al., 1992). Furthermore, BL has been shown to act as inductor of validamycin production in *Streptomyces hygroscopicus* 5008 (Tan et al., 2013) and bitespiramycin production in *Streptomyces spiramyceticus* (Gao et al., 2019). EMSAs were carried out with lysates of *SLspbR-OE* and *SLpapR3-OE* together with P*papR5* and *SLpapR5-OE* with P*papR1* as reported above. In addition to the EMSA samples containing the GBLR lysate and the respective test promoter region, increasing concentrations (1, 2, and 4 μl) of BL and GDL were added, respectively. EMSA analysis revealed that the addition of 1 μl BL to the GBLR test samples eliminated the presence of the shifted bands in samples with SpbR, PapR3, and PapR5, respectively, whereas addition of up to 4 μl GDL did not (Figure 2C). This suggests that BL, but not GDL, acts as artificial ligand for the pristinamycin-specific GBLRs. Since addition of BL prevented the GBLR-DNA binding ability of SpbR, PapR3, and PapR5, this may indicate that the pristinamycin-specific GBLR-like regulators act as “real” GBLRs. GBLs are reported to be highly specific for their cognate GBLRs. The lactone ring is a structural characteristic and essential for the functionality of the GBLs. The synthetic GBLs BL and GDL used here only differ in the additional C6-fatty acid side chain present in GDL (Figure 2A vs. Figure 2B). This suggests that the *in vitro* binding of GDL to the receptors is prevented due to the long fatty acid side chain.

#### Addition of BL Induces Pristinamycin Production in *S. pristinaespiralis*

**FIGURE 3:**
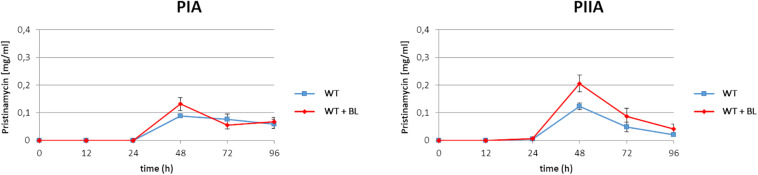
Pristinamycin PIA and PIIA production process of *S. pristinaespiralis* WT with (red) and without addition of BL (blue). Production curves are shown as the averages of three different experiments conducted in triplicate. Error bars indicate standard deviations.

As found from the above mentioned EMSA studies, BL serves as an artificial ligand for the pristinamycin-specific GBLR-like regulators (Figure 2). In a previous study it has been shown that external addition of synthetic GBLs to the *S. pristinaespiralis* culture induces pristinamycin biosynthesis (Paquet et al., 1992). Furthermore, addition of BL induced validamycin and bitespiramycin production before (Tan et al., 2013; Gao et al., 2019). Thus, it can be expected that addition of the artificial ligand BL to the *S. pristinaespiralis* culture induces pristinamycin production. To investigate whether addition of synthetic BL has an inducing effect on pristinamycin biosynthesis, the *S. pristinaespiralis* WT was grown in pristinamycin production medium and 2 mM BL was added to the culture at the expected exponential growth phase at time point 12 h. Cell culture samples were harvested at different time points (12, 24, 48, 72, and 96 h) and treated for pristinamycin extraction and analysis by HPLC. *S. pristinaespiralis* WT samples without the addition of BL served as a reference. HPLC analysis revealed a clearly increased pristinamycin production profile for the BL-induced samples in comparison to the non-induced samples (Figure 3). At 48 h, the BL induced strains produced ∼30% more IA (PIA) (0.13 mg/ml) and ∼40% pristinamycin IIA (PIIA) (0.21 mg/ml) than the non-induced strains (0.09 mg/ml PIA and 0.12 mg/ml PIIA, respectively) (Figure 3). Thus, it could be shown that the addition of synthetic BL induces pristinamycin production resulting in higher concentrations of pristinamycin.

#### Pristinamycin Is Not an Effector for SpbR, PapR3, and PapR5

**FIGURE 4:**
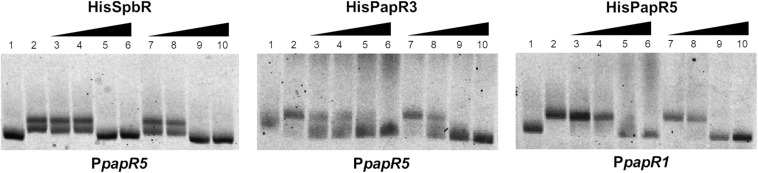
EMSAs performed in 2% agarose gel with cell lysate samples from *SLspbR-OE* **(left)**, *SLpapR3-OE* **(middle)** with P*papR5* and *SLpapR5-OE* **(right)** with P*papR1* in the presence of pristinamycin. 1, promotor DNA; 2, promotor DNA + cell lysate from GBLR overexpression sample; 3–6, addition of increasing concentration (1, 2, 4, 6 μl) of pristinamycin; 7–10, addition of increasing concentration (1, 2, 4, 6 μl) of CH_3_OH:CH_2_Cl_2_ (1:1) as control.

Since addition of synthetic BL prevented formation of the GBLR-DNA interactions in EMSAs as mentioned above, it can be speculated that SpbR, PapR3, and PapR5 act as “real” GBLRs in *S. pristinaespiralis*. To find out whether protein-DNA interaction is influenced by pristinamycin as a potential effector molecule, EMSAs were carried out in the presence of increasing concentrations (1, 2, 4, 6 μl) of pure pristinamycin (50 mg/ml). Pristinamycin was added as a mixture of PIA and PIIA (1:1) dissolved in a solution of 1:1 methanol (CH_3_OH), dichloromethane (CH_2_Cl_2_). Comparable concentrations of a 1:1 methanol/dichloromethane solution were added to each EMSA approach as negative control. EMSA assays showed that addition of pristinamycin did not affect the GBLR-DNA binding pattern (Figure 4, lines 3–6). Only samples with 4–6 μl pristinamycin prevented the formation of a shifted band (Figure 4, lines 5, 6), however, this was due to high concentrations of the methanol/dichloromethane solution since also no shifted band was observed in the corresponding negative control samples (Figure 4, lines 9, 10). These data showed that pristinamycin does not serve as an effector molecule for the GBLR-like regulators SpbR, PapR3, and PapR5 and indicate that the pristinamycin-specific GBLRs do not function as pGBLRs.

#### *S. pristinaespiralis* Culture Extracts Contain Endogenous Component(s) That Affect the GBLR-Like Regulator Binding

**FIGURE 5:**
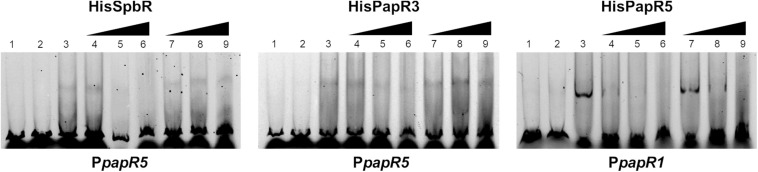
EMSAs performed in 5% acrylamide gel with cell lysate samples from *SLspbR-OE* **(left)**, *SLpapR3-OE* **(middle)** with P*papR5* and *SLpapR5-OE* **(right)** with P*papR1* in the presence of *S. pristinaespiralis* WT culture extract. 1, promotor DNA; 2, promotor DNA + *SLpGM190* lysate; 3, promotor DNA + cell lysate from GBLR overexpression sample; 4–6, addition of increasing concentration (1, 2, 4 μl) of *S. pristinaespiralis* WT culture extract (36 h); 7–9, addition of increasing concentration (1, 2, 4 μl) of methanol as control.

The above mentioned EMSAs suggest that synthetic BL but not pristinamycin is able to bind as ligand to the pristinamycin-specific GBLR-like regulators. To investigate if the pristinamycin-producing strain *S. pristinaespiralis* owns components which affect the regulator-DNA binding activity of SpbR, PapR3, and PapR5, EMSAs were performed in the presence of *S. pristinaespiralis* culture extracts. For this purpose, the *S. pristinaespiralis* wild-type (WT) was grown in pristinamycin production medium and culture supernatant samples were harvested at different time points (30, 36, 42, 48 h). Cell culture samples were extracted and methanolic culture extracts were analyzed by HPLC for pristinamycin production. The different cultivation time points were chosen to cover different phases of antibiotic production: before the start of antibiotic biosynthesis (30 h), during pristinamycin production (36, 42 h) and at the maximum of pristinamycin production (48 h) (Mast et al., 2015). According to Paquet et al. (1992) effector production in *S. pristinaespiralis* begins at about 3 h before the initiation of pristinamycin production. Thus, at 36 h both, effector molecules and pristinamycin, should be present in the prepared extracts. In order to investigate whether endogenous *S. pristinaespiralis* factors bind to the regulatory proteins SpbR, PapR3, and PapR5, EMSAs were performed in the presence of *S. pristinaespiralis* WT extracts in a similar composition as described above. Data are shown for sample time point 36 h in Figure 5 and for residual cultivation time points in Supplementary Figure S2. Shifts with methanol served as negative control (Figure 5, lines 7–9). EMSA analysis showed that formation of a shifted band is prevented upon addition of 2–4 μl *S. pristinaespiralis* WT extract in samples with SpbR and PapR3 together with the promoter region P*papR5* and samples with PapR5 and P*papR1* (Figure 5, lines 4–6). Overall, these data exhibit that the *S. pristinaespiralis* WT culture extract contains endogenous components that serve as effectors for the pristinamycin-specific GBLR-like regulators SpbR, PapR3, and PapR5.

**FIGURE 6:**
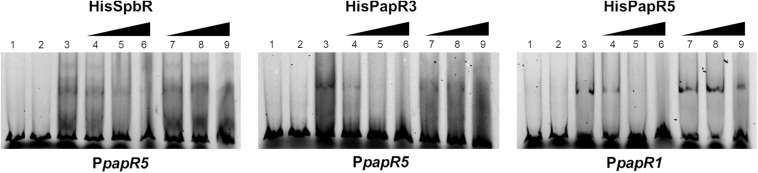
EMSAs performed in 5% acrylamide gel with cell lysate samples from *SLspbR-OE* **(left)**, *SLpapR3-OE* **(middle)** with P*papR5* and *SLpapR5-OE* **(right)** with P*papR1* in the presence of *S. pristinaespiralis papR2::apra* culture extract. 1, promotor DNA; 2, promoter + *SLpGM190* lysate; 3, promotor DNA + cell lysate from GBLR overexpression sample; 4–6, addition of increasing concentration (1, 2, 4 μl) of *S. pristinaespiralis papR2::apra* culture extract (36 h); 7–9, addition of increasing concentration (1, 2, 4 μl) of methanol as control.

To investigate if the effectors, which cause the prevention of the regulator-DNA interaction are associated to the biosynthesis products of the pristinamycin pathway, EMSAs were performed in the presence of extract samples from the pristinamycin-deficient mutant *S. pristinaespiralis papR2::apra*. In *papR2::apra* the essential SARP-type regulator gene *papR2* is deleted, which causes a complete loss of pristinamycin production (Mast et al., 2015; Supplementary Table S1). Strain cultivation, compound extraction and EMSA assays have been carried out in the same way as described for WT culture extract samples. EMSA assays revealed that addition of culture extracts from the *papR2::apra* mutant strain prevented the formation of shifted bands in the respective GBLR-regulator/DNA samples in a similar manner as observed for the WT extract samples [Supplementary Figure S6, lines 4–6 (36 h); Supplementary Figure S3 (30, 42, 48 h)]. As pristinamycin-deficient culture extracts prevented shift formation, it can be concluded that also the *papR2::apra* mutant harbors the endogenous components that serve as ligands for the pristinamycin-specific GBLR-like regulators. This shows that pristinamycin does not act as functional ligand for SpbR, PapR3, and PapR5. This is to be expected since shifted bands would have been remained if pristinamycin served as a functional effector for the GBLR regulators, which however is absent in the *papR2::apra* mutant. This is in line with the observation that pure pristinamycin does not act as ligand for the pristinamycin-specific GBLR regulators and depicts that SpbR, PapR3, and PapR5 do not act as pGBLRs in *S. pristinaespiralis*.

### Identification of the Potential Pristinamycin Effector Biosynthesis Gene *snbU*

**FIGURE 7:**
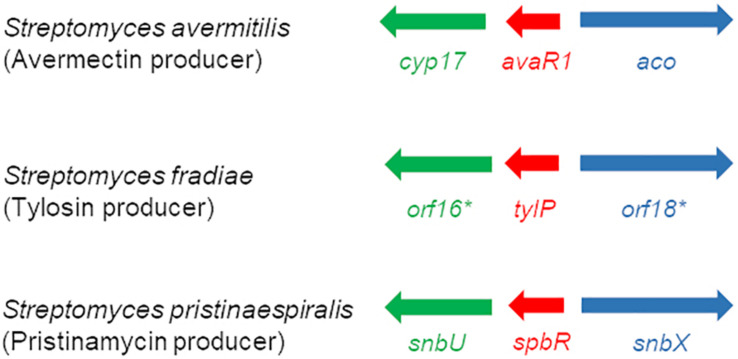
Schematic presentation of the organization of regulatory genes in the avermectin producer *S. avermitilis* **(top)**, the tylosin producer *S. fradiae* **(middle)** (Kitani et al., 2011), and the pristinamycin producer *S. pristinaespiralis* **(bottom)** (modified according to Kitani et al., 2011). Homologous genes are labeled with the same color: GBLR gene (red), cytochrome P450 gene (green), and acyl-CoA oxidase gene (blue).

EMSA assays showed that synthetic GBL but not pristinamycin-associated samples affect the DNA binding activity of SpbR, PapR3, and PapR5, which would hint for a GBL-like molecule as GBLR-interacting ligand in *S. pristinaespiralis*. So far, the chemical structure of the GBLR-interacting ligand molecule as well as its encoding biosynthetic gene(s) are unknown for *S. pristinaespiralis*. Well-known GBL biosynthesis genes are *afsA* and *scbA* from *S. griseus* and *S. coelicolor*, respectively. Both genes encode for fatty acid synthase-like enzymes, whereby AfsA is responsible for the biosynthesis of the A-factor in *S. griseus* (Ando et al., 1997) and ScbA catalyzes the synthesis of the SCBs in *S. coelicolor* (Takano et al., 2008). No *afsA/scbA*-like gene could be identified for *S. pristinaespiralis* (Mast et al., 2015). In *S. pristinaespiralis* the GBLR-like genes *spbR, papR3*, and *papR5* are part of a regulatory region localized at the right border of the pristinamycin biosynthesis gene cluster (Mast et al., 2011). Between the genes *papR5* and *spbR* the gene *snbU* is located, which encodes for a putative cytochrome P450 monooxygenase SnbU (Mast et al., 2015; Figure 7). SnbU shows amino acid sequence similarity to Orf16^∗^ (64% identity, 75% similarity) from the tylosin producer *Streptomyces fradiae* and Cyp17 (63% identity, 74% similarity) from the avermectin producer *Streptomyces avermitilis.* Both proteins have been suggested to be involved in effector biosynthesis in the respective antibiotic producers (Bignell et al., 2007; Kitani et al., 2011). In *S. fradiae orf16*^∗^ is located upstream of the gene *tylP* encoding the tylosin GBLR regulator TylP and *orf18*^∗^ encoding a putative acyl-CoA oxidase (Figure 7). In *S. avermitilis cyp17* shows a similar genetic localization and is found upstream of the gene *avaR1* encoding the GBLR regulator AvaR1, and the gene *aco* encoding an acyl-CoA oxidase involved in avenolide effector synthesis (Kitani et al., 2011; Figure 7). Due to the amino acid sequence similarities between Orf16^∗^ and Cyp17 with SnbU and the comparable genetic organization of the coding genes within the respective antibiotic biosynthetic gene clusters, it can be assumed that SnbU is involved in the biosynthesis of the GBLR-interacting ligand(s) in *S. pristinaespiralis*. Thus, we analyzed the *snbU* gene in respect to its influence on pristinamycin production and GBLR-DNA interactions (see below).

#### Inactivation of *snbU* Has Regulatory Influence on Pristinamycin Production

**FIGURE 8:**
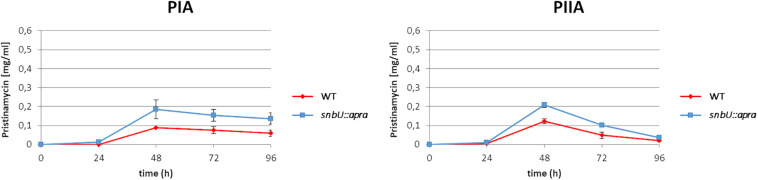
Pristinamycin production [PIA **(left)**, PIIA **(right)** of *S. pristinaespiralis* WT (red) and the *snbU::apra* mutant (blue)]. Production curves are shown as the averages of three different experiments conducted in triplicate. Error bars indicate standard deviations.

In order to investigate the function of the putative effector biosynthesis gene *snbU*, the gene was inactivated by insertion of an apramycin resistance cassette (Apr^R^) and the pristinamycin production of the resulting *snbU::apra* mutant (Supplementary Table S1) was compared with that of the *S. pristinaespiralis* WT. For this purpose, the *snbU::apra* mutant and the WT were grown in pristinamycin production medium. Samples were taken at different time points (24, 48, 72, and 96 h) and pristinamycin extracts were analyzed by HPLC. The HPLC analysis showed that PIA and PIIA production was increased in the *snbU::apra* mutant in comparison to the WT. At the production maximum at 48 h, the *snbU::apra* mutant produced twice as much PIA (0.19 mg/ml) and ∼40% more PIIA (0.21 mg/ml) than the WT (0.09 mg/ml PIA and 0.12 mg/ml PIIA, respectively) (Figure 8). Complementation of *snbU::apra* with the *snbU* expression construct pGM190/snbU resulted in a pristinamycin production profile as similarly, observed for the WT strain (Supplementary Figure S4). These data show that inactivation of *snbU* results in an increased pristinamycin production. Thus, it can be concluded that SnbU has a regulatory influence on pristinamycin biosynthesis in *S. pristinaespiralis*.

### Inactivation of *snbU* Prevents Production of GBLR-Binding Endogenous Components

**FIGURE 9:**
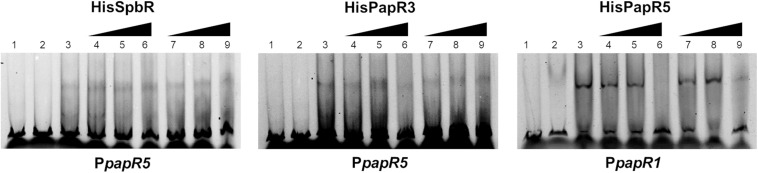
EMSAs performed in 5% acrylamide gel with cell lysate samples from *SLspbR-OE* **(left)**, *SLpapR3-OE* **(middle)** with P*papR5* and *SLpapR5-OE* **(right)** with P*papR1* in the presence of *S. pristinaespiralis snbU::apra* culture extract. 1, promotor DNA; 2, promotor DNA + *SLpGM190* lysate; 3, promotor DNA + cell lysate from GBLR overexpression sample; 4–6, addition of increasing concentration (1, 2, 4 μl) of *S. pristinaespiralis snbU::apra* culture extract (36 h); 7–9, addition of increasing concentration (1, 2, 4 μl) of methanol as control.

In the above mentioned experiments it has been shown that *S. pristinaespiralis* culture extracts contain endogenous effectors that abolish the regulatory binding of the pristinamycin-specific GBLRs to their target promoters. Furthermore, it has been displayed that SnbU has a regulatory influence on pristinamycin production. To analyze if *snbU* is involved in the biosynthesis of the GBLR-interacting ligand EMSAs were performed with SpbR, PapR3, and PapR5 in the presence of culture extract samples from the *S. pristinaespiralis snbU::apra* mutant. EMSAs were carried out as mentioned above and are shown representatively for *snbU::apra* culture sample time point 36 h. EMSA analysis showed that addition of culture extracts from the *snbU::apra* mutant did not prevent shift formation in any of the tested GBLR-promoter samples [Figure 9, lines 4–6 (36); Supplementary Figure S5 (30, 42, 48 h)]. This is different from the observation obtained from EMSAs in the presence of *S. pristinaespiralis* WT and *papR2::apra* mutant culture extracts, which did prevent shift formation (Figure 9 vs. Figures 5, 6, respectively). This finding shows that culture extracts from *S. pristinaespiralis* WT and *papR2::apra* contain endogenous factors that function as ligands for the pristinamycin-specific GBLR regulators, which are absent in the *snbU::apra* mutant. Thus, it can be concluded that SnbU is involved in the biosynthesis of the GBLR-interacting ligand molecule in *S. pristinaespiralis*.

## Discussion

Antibiotic biosynthesis is known to be governed by quorum sensing-like systems involving GBLs as signaling molecules. Nodal points of regulation are represented by GBLR regulators, which sense the abundance of GBLs and transmit the effector signal to the biosynthesis output. In addition to the well-known “real” GBLRs, which bind GBLs as ligands there are also several examples of “pseudo” GBLRs binding antibiotics or antibiotic intermediates as ligands (Biarnes-Carrera et al., 2015; Niu et al., 2016; Table 1). In *S. pristinaespiralis* three GBLR-like regulators are known to be involved in regulation of pristinamycin biosynthesis (Mast et al., 2015). So far, it was not known if the regulators act as real GBLRs or pGBLRs. To elucidate the function of the respective GBLR-like regulators in *S. pristinaespiralis*, EMSAs were carried out in this study, which showed that all three pristinamycin-specific GBLR-like regulators, SpbR, PapR3, and PapR5, belong to the group of “real” GBLRs since regulator-DNA interactions were influenced by GBL-associated samples but not by pristinamycin-specific samples.

It remains to be elucidated if all three GBLRs, SpbR, PapR3, and PapR5, sense the same chemical ligand or if structurally different effector molecules are recognized and how this translates to the working model of the pristinamycin hierarchical signaling cascade. Additional information on the pristinamycin-specific GBLRs has been gained by bioinformatic analysis with the I-TASSER software, which allows to perform *in silico* protein structure and function predictions (Yang et al., 2014). I-TASSER enables to predict three-dimensional structural models and potential ligands of protein molecules based on amino acid sequences. I-TASSER analysis with the amino acid sequences of SpbR, PapR3, and PapR5 revealed nucleic acids as a potential ligand for all three GBLR-like proteins (Supplementary Figure S6 and Supplementary Table S2). Furthermore, small, unpolar, mostly aromatic-like metabolites have been identified as potential ligands (Supplementary Figure S6 and Supplementary Table S2), whereby SpbR and PapR5 showed more overlap in the suggested ligand types than PapR3 (Supplementary Table S2). This may indicate that SpbR and PapR5 sense the same or a similar ligand substance, which is different from the effector molecule recognized by PapR3. The fact that no GBL- or avenolide-like molecule has been proposed by the I-TASSER program as potential ligand might be because no GBLRs from antibiotic-producing actinomycetes with their cognate ligands are deposited in the I-TASSER data base.

The structure of the pristinamycin-specific GBLR-interacting ligand molecule(s) as well as the encoding biosynthetic gene(s) are unknown for *S. pristinaespiralis* so far. In this work, the gene *snbU*, which codes for a putative cytochrome P450 monooxygenase is described to be involved in the biosynthesis of the pristinamycin-specific GBLR-interacting ligand(s). Surprisingly, inactivation of *snbU* in *S. pristinaespiralis* led to an increased pristinamycin production profile. In contrast, inactivation of genes involved in antibiotic effector biosynthesis in other streptomycetes, often result in loss of antibiotic production or a decrease in production yields. For example, inactivation of *afsA*, which is responsible for A-factor biosynthesis in *S. griseus*, abolished streptomycin production completely (Horinouchi, 2002). However, also antibiotic overproduction has been reported for producers with inactivated GBL biosynthesis genes. For example, in *S. coelicolor*, the deletion of the GBL synthase gene *scbA*, which abolished the biosynthesis of the SCB1 effector molecule resulted in precocious and increased production of ACT and RED (Takano et al., 2008). The cognate GBLR for the SCB1 effector in *S. coelicolor* is ScbR, which represses its own transcription, as well as that of *scbA*. For *S. coelicolor* it has been proposed that deletion of *scbA* abolishes SCB1 production, which results in a basal level of ligand-free ScbR, which itself is suggested to repress the synthesis of a hierarchical subordinate transcriptional repressor of antibiotic biosynthesis giving rise to overproduction of ACT and RED (Takano et al., 2008). Similarly, the pristinamycin overproducing effect of the *snbU::apra* mutant might be explained by the complex hierarchical organization of the regulatory signaling cascade. In this respect inactivation of *snbU* would cause loss of effector synthesis resulting in low levels of ligand-free GBLR, e.g., SpbR, which would be inefficient for repressing the transcription of the hierarchical subordinate repressor gene *papR5*, resulting in increased pristinamycin production levels. This would be in accordance with the observation that deletion of *papR5* in *S. pristinaespiralis* results in dramatically increased pristinamycin production (Mast et al., 2015). Cytochrome P450 monooxygenases have been suggested before to be involved in antibiotic effector synthesis. In *S. fradiae* and *S. avermitilis, orf16^∗^* and *cyp17* both encode for predicted cytochrome P450 monooxygenases, respectively, and are suggested to participate in effector synthesis in the respective antibiotic producers. *snbU, orf16^∗^* and *cyp17* are each located downstream of a GBLR-type regulator gene and a gene encoding an acyl-CoA oxidase (Figure 7). Also, the acyl-CoA oxidase encoding genes *orf18*^∗^ and *aco* from *S. fradiae* and *S. avermitilis*, respectively, have been reported to be involved in effector biosynthesis, whereas the encoded GBLR proteins serve as the ligand receptors. Inactivation of *orf16*^∗^ and *orf^∗^18* in *S. fradiae* resulted in a reduction of tylosin production to 40–60% of the WT production level and loss of production of the TylP-binding ligand (Bignell et al., 2007). Thus, it was concluded that both genes are involved in the synthesis of the TylP-associated ligand. However, so far the TylP ligand has not been identified and the involvement of *orf16*^∗^ and *orf18*^∗^ in ligand synthesis has not been demonstrated (Zou et al., 2014). Inactivation of *aco* in *S. avermitilis* resulted in a reduction of avermectin production to ∼6% of wild-type levels and loss of avenolide effector synthesis (Kitani et al., 2011). Aco has been proposed to introduce the double bond into the C2–C3 position of the avenolide effector from *S. avermitilis* (Kitani et al., 2011; Supplementary Figure S7). A putative *aco*-homologous gene, *snbX* (accession number: MT563328), has been identified downstream of *spbR* in the course of preparing this manuscript and is not experimental part of this study (Figure 7). The gene was identified based on a newly obtained PacBio genome sequence from *S. pristinaespiralis* Pr11 (accession number CP059696). The predicted gene product SnbX shows homology to the putative acyl-CoA oxidases Orf18 (46% identity, 58% similarity) from *S. fradiae* and Aco (53% identity, 64% similarity) from S. *avermitilis* and thus may also be involved in the biosynthesis of the GBLR-interacting ligand(s) in *S. pristinaespiralis*. Due to the genetic similarity of the mentioned effector synthesis genes and the experimental data on the avenolide effector, it can be expected that the pristinamycin-specific ligand(s) may represent a signaling molecule of the avenolide type. This is also in line with the observation that alkaline treatment of GBLR-samples in the presence of *S. pristinaespiralis* WT extracts did not influence the shift performance (Supplementary Figure S8), as similarly, observed for respective samples with the avenolide effector (Kitani et al., 2011). Instead, GBLs are known to be sensitive to alkaline treatment but resistant to treatments with protease, heat and acid (Du et al., 2011). Thus, also these data support the hypothesis that the signaling molecule from *S. pristinaespiralis* belongs to the avenolide type of autoregulators. So far, it cannot be excluded that additional genes/enzymes are involved in effector synthesis in *S. pristinaespiralis*. The pristinamycin gene region contains three genes of unknown function – *snbS*, *snbW*, and *snbV.* The genes are located at the right border of the cluster next to the “regulatory operon” upstream of *papR3* (Mast et al., 2011). *snbS* encodes a putative methylmalonyl-CoA decarboxylase, while *snbW* and *snbV* carry the information for hypothetical proteins. In order to investigate their involvement in pristinamycin biosynthesis and/or regulation *snbS*, *snbW*, and *snbV* will be inactivated in ongoing experiments. Furthermore, it would be important to isolate and solve the chemical structure of the GBLR-interacting molecule from *S. pristinaespiralis*, which so far was hindered by low ligand concentrations. However, the availability of a ligand-free *snbU::apra* mutant, which can serve as a reference in analytic analysis, may facilitate structure elucidation approaches in future studies.

In this work, we demonstrated that SpbR, PapR3, and PapR5 belong to the class of real GBLRs, which most likely bind avenolide-type effector molecules as ligands. The gene *snbU* encoding a predicted cytochrome P450 monooxygenase has been demonstrated to be involved in the biosynthesis of the GBLR-interacting ligand.

## Materials and Methods

### Bacterial Strains, Plasmids, and Cultivation Conditions

The bacterial strains and plasmids used in this study are listed in Supplementary Table S1. For routine cloning strategies, *Escherichia coli* NovaBlue (Novagen) was used. *E. coli* strains were grown in Luria-Bertani (LB) medium (Maniatis et al., 1989) at 37°C, supplemented with kanamycin, apramycin, or ampicillin (50, 100, or 150 μg/ml, respectively) when appropriate. *S. pristinaespiralis* Pr11 (Aventis Pharma) was used for the generation of the *snbU* insertion mutant. For cultivation and harvesting of genomic DNA, *Streptomyces* strains were grown in 100 ml of S-medium (Kieser et al., 2000) in 500 ml Erlenmeyer flasks (with steel springs) on an orbital shaker (180 rpm) at 28°C. Kanamycin and apramycin (50 and 100 μg/ml, respectively) were added to liquid cultures when required. For pristinamycin production analysis, *S. pristinaespiralis* strains were cultivated as reported previously (Mast et al., 2011). For EMSA analysis, the *S. pristinaespiralis* strains were sampled at different time points after 30, 36, 42, and 48 h. 100 ml of *S. pristinaespiralis* culture was extracted with 100 ml ethyl acetate for 20 min and concentrated completely *in vacuo*. The extract was then redissolved in appropriate volumes of methanol (2 ml methanol for 100 ml extraction volume). *Streptomyces lividans* T7 (Fischer, 1996; Supplementary Table S1) was used for protein expression experiments. *S. lividans* strains were GBLR expression strains *SLpapR3-OE, SLpapR5-OE, SLspbR-OE, and SLpGM190* (control) were cultivated in yeast extract-malt extract (YEME) medium as reported before (Mast et al., 2015).

### Molecular Cloning

DNA manipulation procedures were performed as described previously for *E. coli* (Maniatis et al., 1989) and for *Streptomyces* (Kieser et al., 2000). The primers used for PCR were obtained from Eurofins MWG Biotech AG (Ebersberg, Germany) and are listed in Supplementary Table S1.

### Expression of the Pristinamycin Regulators

For protein expression, *S. lividans* GBLR expression strains *SLpapR3-OE, SLpapR5-OE, SLspbR-OE*, and *SLpGM190* (control) were constructed and cultivated as reported before (Mast, 2008; Mast et al., 2015; Supplementary Table S1).

### Electromobility Shift Assays (EMSAs)

DNA fragments of the upstream regions of the regulatory genes *papR1* (P*papR1*, 240 bp) and *papR5* (P*papR5*, 260 bp) were amplified by PCR from genomic DNA of *S. pristinaespiralis* Pr11 with primers listed in Supplementary Table S1. Promoter DNA amplificates included a 16 bp Cy5 adapter sequence, each at the 3′ and 5′ end, which was added via the respective primer sequences. The generated amplificates were used as templates in a second PCR approach together with a Cy5 primer (Supplementary Table S1) in order to conduct Cy5 labeling of the promoter regions. For EMSAs, cell lysates from *S. lividans* strains were used, which overexpressed the respective His-tagged GBLR-like regulators together with 2 ng of the respective Cy5-labeled promotor region, P*papR5* or P*papR1*.

DNA-regulator binding reactions were performed at room temperature as described previously (Mast et al., 2015). The samples were loaded onto a 2% agarose gel for testing the specificity of regulator-DNA binding and effect of pristinamycin addition. For EMSAs with synthetic GBLs and *S. pristinaespiralis* culture extracts 5% acrylamide gels were used. EMSAs were carried out with variable concentrations of regulator lysate sample as reported before (Mast et al., 2015). To verify the specificity of the regulator-DNA binding, an excess of unlabeled, specific target DNA (P*papR1* or P*papR5*) and non-specific DNA (*hrdB*), respectively, was added to the EMSA mixture as described previously (Mast et al., 2015). DNA bands were visualized by fluorescence imaging using a Typhoon Trio^TM^ Variable Mode Imager (GE Healthcare). All EMSAs were carried out at least three times independently. Pure BL (0.948 g/ml) and GDL (1.12 g/ml) were purchased by Sigma-Aldrich. Pure pristinamycin (50 mg/ml), consisting of a 1:1 mixture of PIA and PIIA was obtained from Sanofi-Aventis Pharma.

### Construction of the *S. pristinaespiralis* snbU::*apra* Mutant

For construction of a *snbU* apramycin insertion mutant, a ∼2.6 kb fragment was amplified by PCR using Taq polymerase and primers snbUfw/snbUrev listed in Supplementary Table S1. Cosmid 1/12 DNA served as template, which was obtained from a *S. pristinaespiralis* cosmid library and harbored part of the pristinamycin gene cluster. The PCR-derived ∼2.6 kb amplificate *snbU’* included the 1,320 bp *snbU* coding sequence, as well as a 437 bp up- and a 787 bp downstream region. *snbU’* was subcloned into the *Eco*RV-linearized *E. coli* cloning vector pDrive, resulting in the construct pDrive/snbU.’ snbU’ was isolated from pDrive/snbU’ as *Eco*RI fragment and ligated to the *Eco*RI-restricted *E. coli* vector pK18 (Pridmore, 1987; Supplementary Table S1), resulting in the construct pK18/snbU.’ A 1.5 kb apramycin resistance cassette (Apr^R^) was isolated as an *Eco*RV/*Sma*I fragment from pEH13 (Heinzelmann et al., 2001; Supplementary Table S1) and cloned into the *Nru*I restriction site of *snbU,’* which yielded the final mutagenesis construct pK18/snbUapra, in which the gene *snbU* is inactivated by an apramycin resistance cassette. This construct was transferred to *S. pristinaespiralis* Pr11 by protoplast transformation, followed by a selection for apramycin-resistant and kanamycin-sensitive transformants, resulting in the *S. pristinaespiralis* mutant *snbU::apra*. The *snbU* mutant was complemented by introducing the expression plasmid pGM190/snbU to *snbU::apra*, resulting in strain *snbU::apra* pGM190/snbU (Supplementary Table S1).

### Pristinamycin Analysis

Pristinamycin was detected by HPLC analysis as reported before (Mast et al., 2011).

#### Bioinformatic Analysis

*In silico* protein structure analysis was performed with the I-TASSER software^1^ (Yang et al., 2014) and the amino acid sequences of SpbR, PapR3, and PapR5 (Mast et al., 2011).

## Data Availability Statement

The datasets presented in this study can be found in online repositories. The names of the repository/repositories and accession number(s) can be found in the article/Supplementary Material.

## Author Contributions

FH carried out the EMSA assays and pristinamycin production analysis. AK and FH did the HPLC analyses. YM carried out bioinformatic analyses and designed, supervised, and coordinated the study. FH and YM wrote the manuscript. All authors contributed to the article and approved the submitted version.

## Conflict of Interest

YM was employed by the company Leibniz Institute DSMZ – German Collection of Microorganisms and Cell Cultures GmbH. The remaining authors declare that the research was conducted in the absence of any commercial or financial relationships that could be construed as a potential conflict of interest.

## Footnotes

1 https://zhanglab.ccmb.med.umich.edu/I-TASSER/

## Abbreviations

ACT: actinorhodin
A-factor: ligand from *S. griseus*
Apr^R^: apramycin resistance cassette
ARE: autoregulatory element
BL: 1,4-butyrolactone
EMSA: electromobility shift assays
HPLC: high performance liquid chromatography
HTH: helix-turn-helix
GBL: γ-butyrolactone
GBLR: γ-butyrolactone receptor
pGBLR: pseudo γ-butyrolactone receptor
GDL: γ-decanolactone
LB: Luria-Bertani
p*I*: isoelectric point
PIA: pristinamycin IA
PIIA: pristinamycin IIA
RED: undecylprodigiosin
RT-PCR: Reverse transcriptase-polymerase chain reaction
SARP: *Streptomyces* antibiotic regulatory protein
SCB: ligands from *S. coelicolor*
SVB: ligands from *S. venezuelae*
SpbR: *S. pristinaespiralis* butyrolactone-responsive transcriptional repressor
TetR: Tetracycline repressor protein
VB: virginiae butanolide; ligand from *S. virginiae*
WT: wild-type
YEME: yeast extract, malt extract.
