# Supplementary figures and images for: Investigation of the Autoregulator-Receptor System in the Pristinamycin Producer Streptomyces pristinaespiralis
Source: Front Microbiol. 2020 Sep 30;11:580990. doi: 10.3389/fmicb.2020.580990 (PMC7554373; doi:10.3389/fmicb.2020.580990)

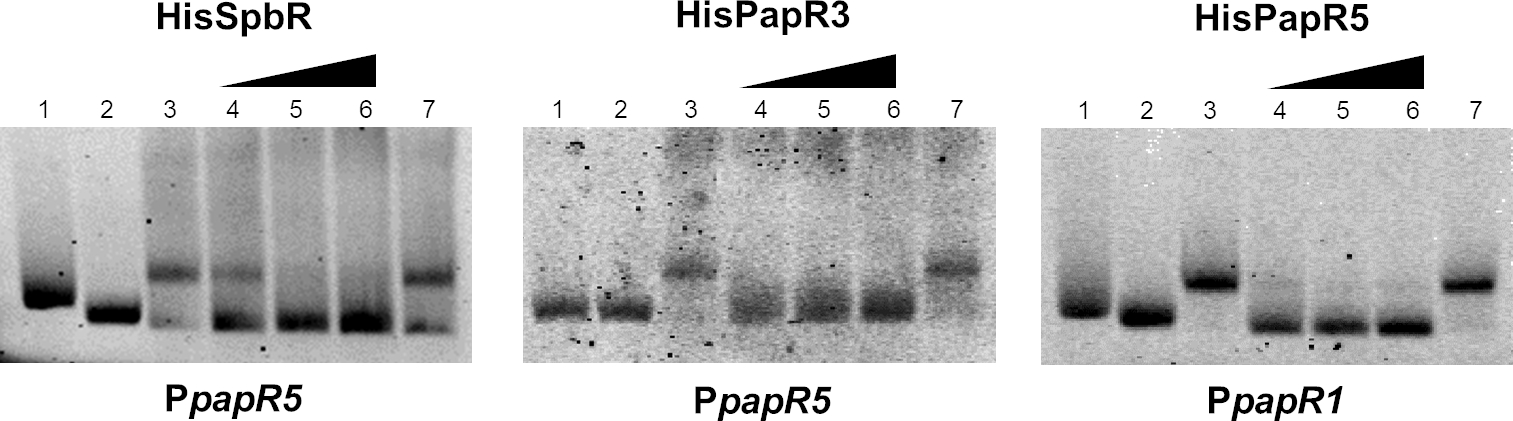

Supplement: Supplementary file 1 [file Image_1.JPEG]

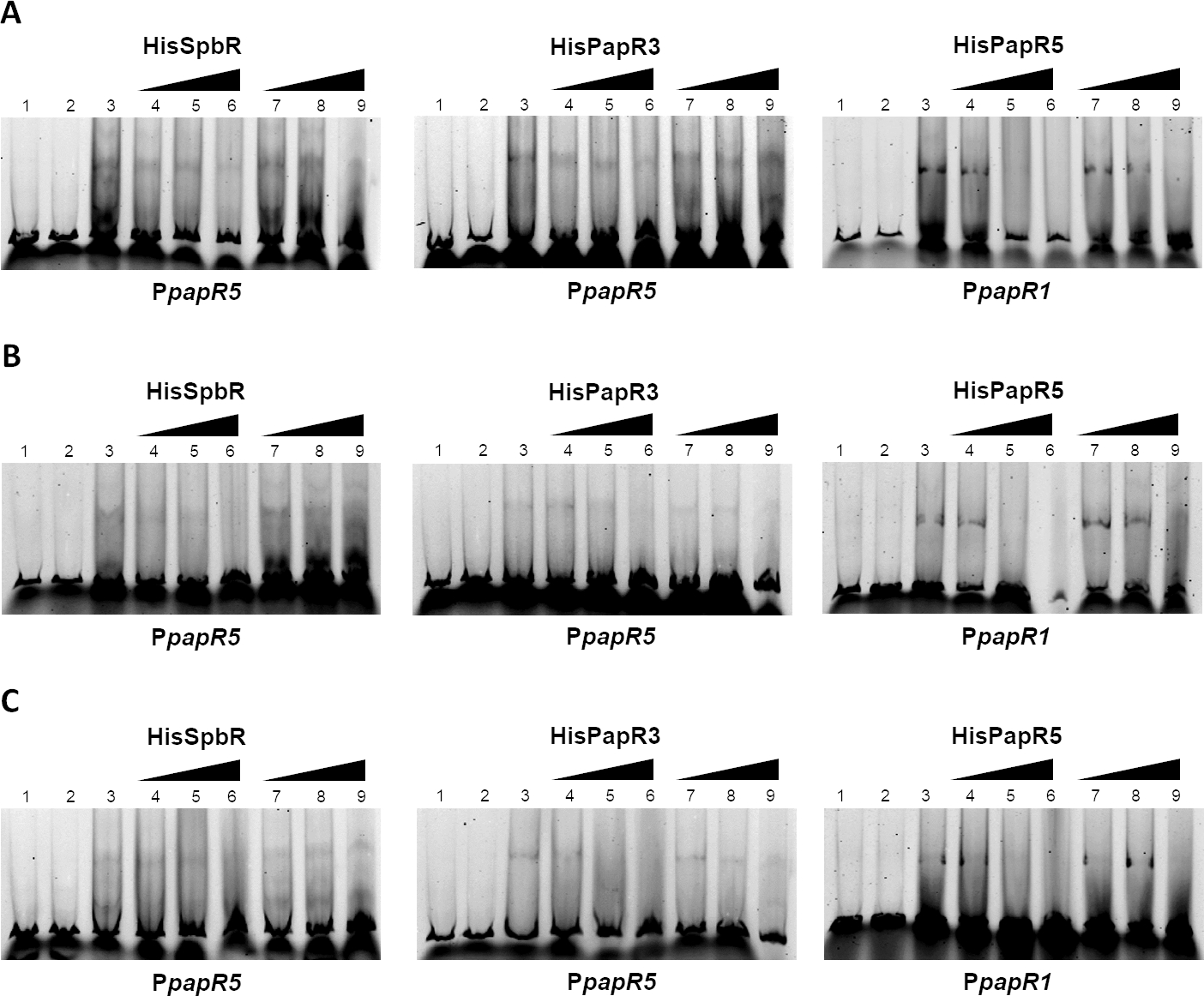

Supplement: Supplementary file 2 [file Image_2.JPEG]

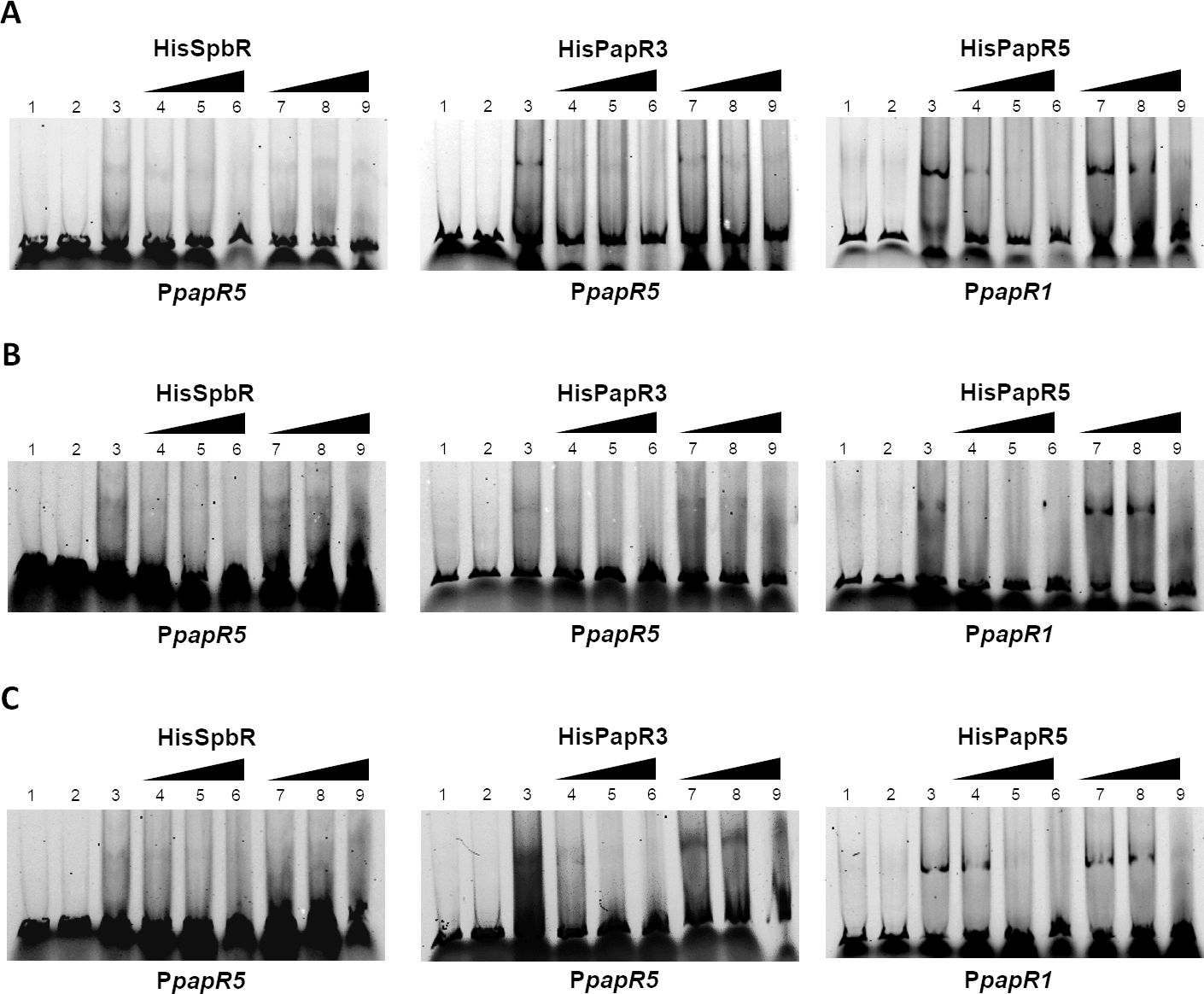

Supplement: Supplementary file 3 [file Image_3.JPEG]

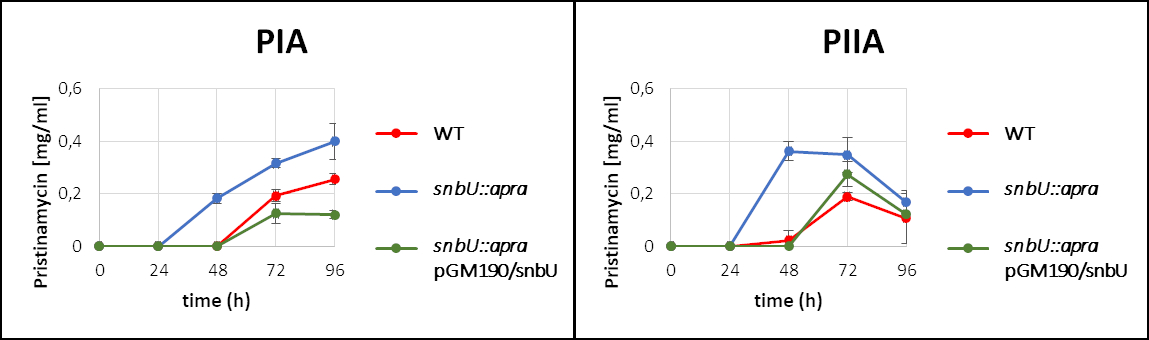

Supplement: Supplementary file 4 [file Image_4.JPEG]

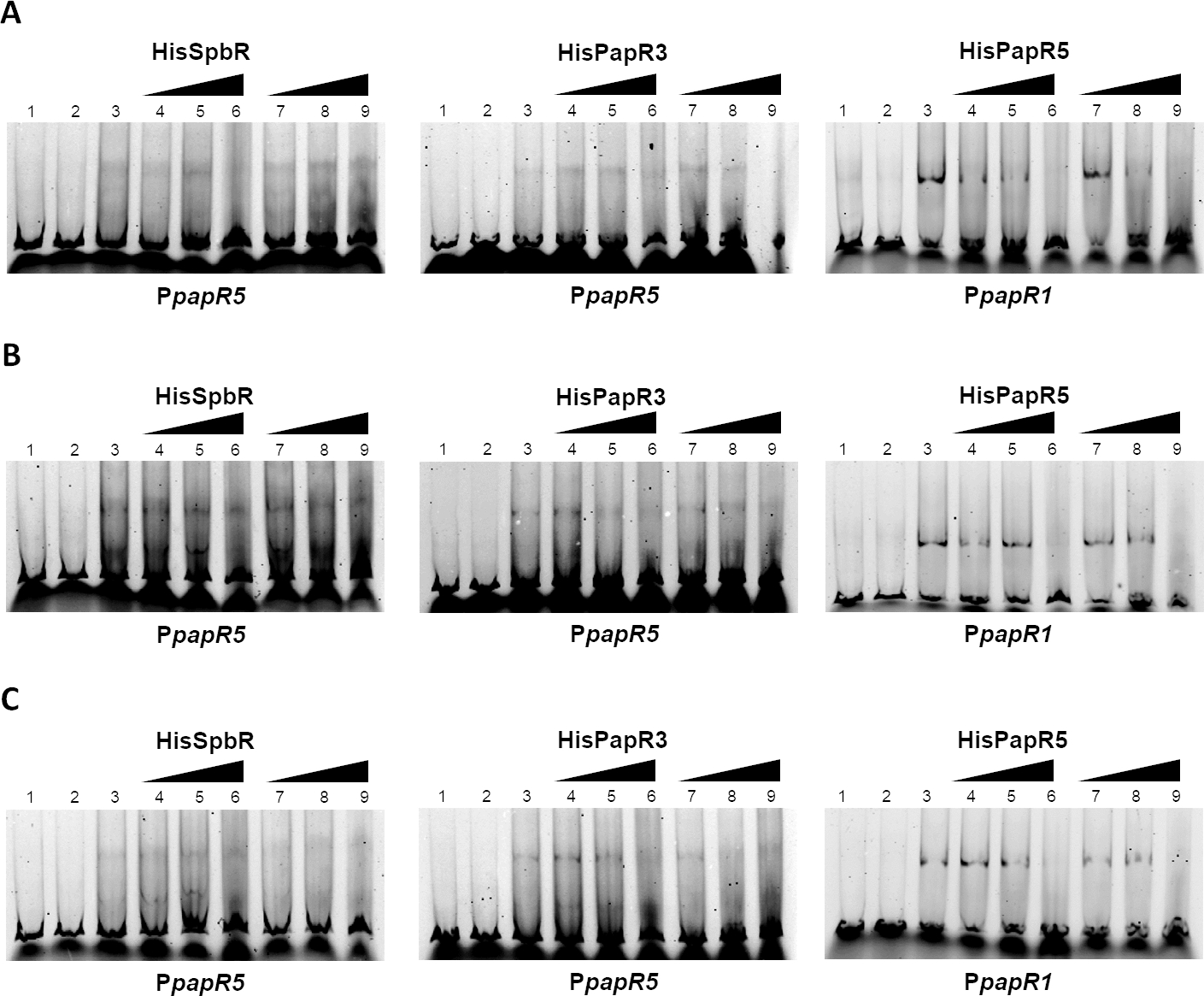

Supplement: Supplementary file 5 [file Image_5.JPEG]

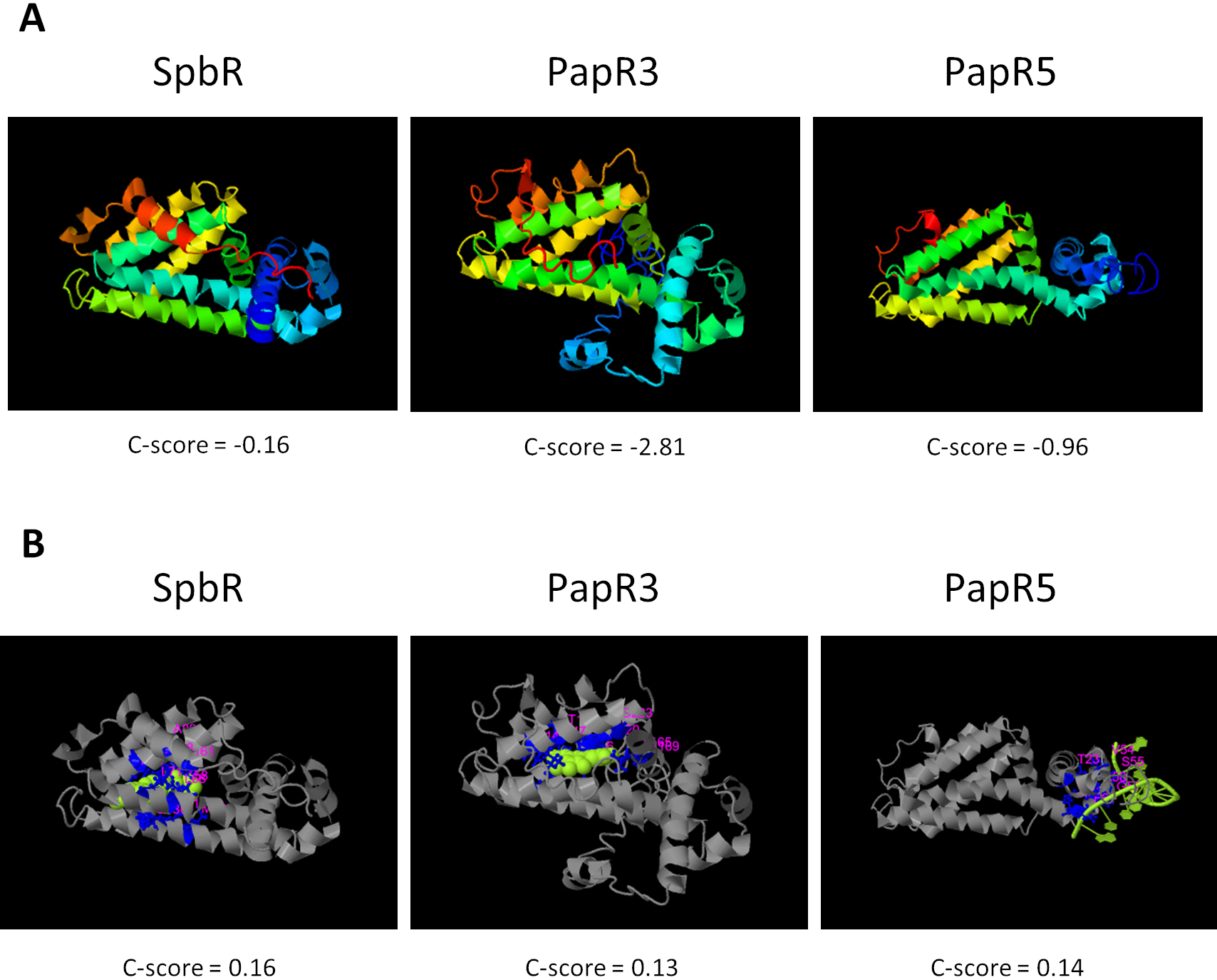

Supplement: Supplementary file 6 [file Image_6.JPEG]

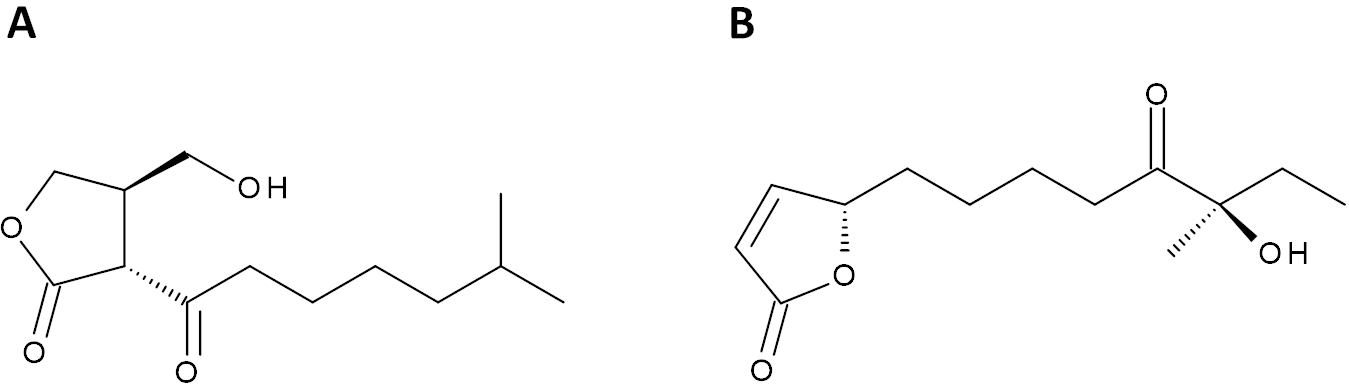

Supplement: Supplementary file 7 [file Image_7.JPEG]

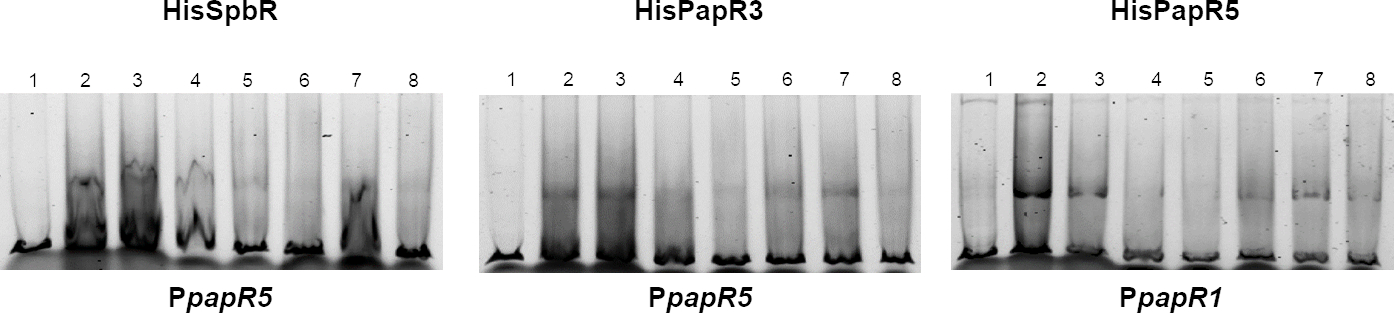

Supplement: Supplementary file 8 [file Image_8.JPEG]
