# Supplementary material for: Investigation of the Autoregulator-Receptor System in the Pristinamycin Producer Streptomyces pristinaespiralis
Source: Front Microbiol. 2020 Sep 30;11:580990. doi: 10.3389/fmicb.2020.580990 (PMC7554373; doi:10.3389/fmicb.2020.580990)
Supplement: Supplementary file 9 [file Data_Sheet_1.PDF]

Supplementary Material

Supplementary Table S1: Bacterial strains, plasmids and primers.

**Bacterial strain**	**Description**	**Source or reference**
E. coli NovaBlue	*endA1, hsdR17* (r_{K12}^{-} m_{K12}^{+}), *supE44, thi-1, recA1, gyrA96, relA1, lac F'[proA^{+}B$^{+,}$ lacI^{q}ZΔM15::Tn10], (tsr)*	Novagen
S. pristinaespiralis		
Pr11	Pristinamycin-producing strain/wild type; natural isolate of *S. pristinaespiralis* ATCC 25486	Aventis Pharma
papR2::apra	*papR2* insertion mutant; gene interruption of *papR2, aac(3)IV*	(Mast et al., 2015)
snbU::apra	*snbU* insertion mutant; gene interruption of *snbU, aac(3)IV*	This work
snbU::apra pGM190/snbU	Complemented *snbU* mutant; *snbU::apra*, pGM190/snbU	This work
S. lividans		
T7	*tsr*, T7 RNA polymerase gene	(Fischer 1996)
SLpGM190	Empty vector control strain; *S. lividans*/pGM190	(Mast et al. 2015)
SLpapR3-OE	PapR3 overexpression strain; *S. lividans*, pGM190/papR3	(Mast et al. 2015)
SLpapR5-OE	PapR5 overexpression strain; *S. lividans*, pGM190/papR5,	(Mast et al. 2015)
SLspbR-OE	SpbR overexpression strain; *S. lividans*, pGM190/spbR,	(Mast et al. 2008)

**Plasmids/Cosmids**	**Description**	**Source or reference**
pDrive	*kan*^{r}*, amp*^{r}*, Plac, lacZ'*	QIAGEN
pK18	pUC-Derivat, *aphII*, *lac*Z'*α*-complementation system	(Pridmore, 1987)
pK18/snbUapra	pK18 derivative, *aphII*, Apr^r, *lac*Z'*α snbU*'	This work
pEH13	pUC21 derivative carrying the 1.8-kb apramycin resistance cassette (*Apr*^{R})	(Heinzelmann et al., 2001)
pGM190	*Streptomyces-E. coli* shuttle vector, *tsr, aphII*, pSG5 derivative, *tipA* promoter shuttle vector	(Wohlleben et al., 2009)
pGM190/papR3	pGM190 derivative, *PtipA, tsr, aphII, hispapR3*	(Mast et al., 2015)
pGM190/papR5	pGM190 derivative, *PtipA, tsr, aphII, hispapR5*	(Mast et al., 2015)
pGM190/spbR	pGM190 derivative, *PtipA, tsr, aphII, hisspbR*	(Mast et al., 2008)
pGM190/snbU	pGM190 derivative, *PtipA, tsr, aphII, snbU*	This work
cosmid 1/12	*Apr*^{R}*, snbR, dec, hyp, hyp, papR3, papR4, papR5, hyp, P450, spbR*	Combinature Biopharm AG

**Primer**	**Primer sequence (5'→3')**	**T_m (°C)**
**For *snbU* mutant construction**		
snbUfw	ACACGCCATGCCCCGCTCCC	58
snbUrev	TTGTTCCTCCGGTCTTCG	61
apra1	GGCATCGCATTCTTCG	57
apra2	GGACCTTGGAGTTGTC	57

**For amplification of promotor regions**		
proppapR1fw	AGCCAGTGGCGATAAGAACGACGGCTGCCTGACCGC	77
propapR1rev	AGCCAGTGGCGATAAGTATGTCGATGTCCATGGCGT	73
proppapR5fw	AGCCAGTGGCGATAAGTCTGTCCCGGTTCCCGGCCC	78
proppapR5rev	AGCCAGTGGCGATAAGATCGCAGCGATCCCCTCACT	75
hrdBfw	GAGTCCGTCTCTGTCATGGCG	62
hrdBrev	TCGTCCTCGTCGGACAGCACG	65
Cy5	AGCCAGTGGCGATAAG	60

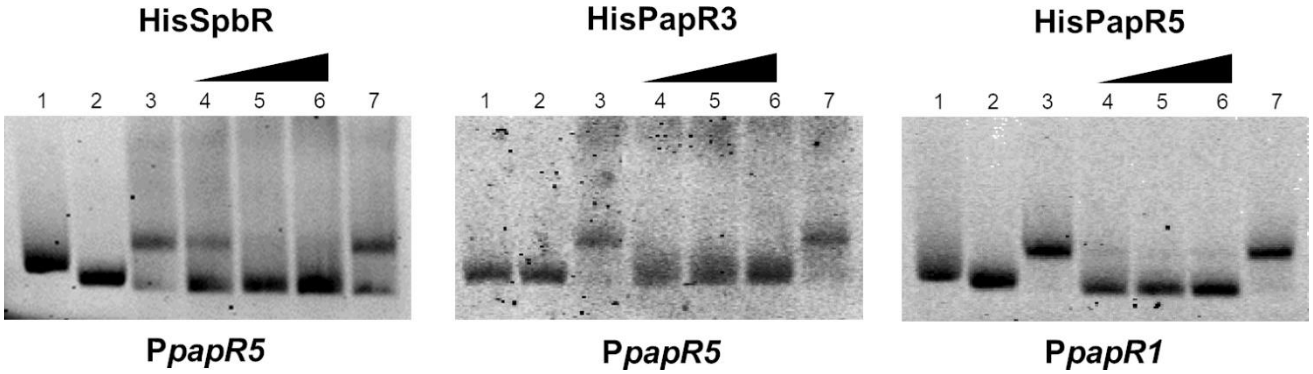

Supplementary Figure S1: EMSAs performed in 2% agarose gels with cell lysate samples from *SLspbR-OE* (left), *SLpapR3-OE* (middle) together with the *papR5* promoter region (P*papR5*), and *SLpapR5-OE* (right) together with the *papR1* promoter region (P*papR1*). 1 = promotor DNA. 2 = promoter DNA + *SLpGM190* lysate, 3 = promotor DNA + cell lysate from GBLR overexpression sample, 4-6 = promotor DNA + cell lysate from GBLR overexpression sample + addition of increasing concentrations (0.3, 1.5 and 3.0 µg) of competitive specific unlabeled promoter DNA, 7 = promotor DNA + cell lysate from GBLR overexpression sample + addition of 2 µg unspecific unlabeled *hrdB* DNA.

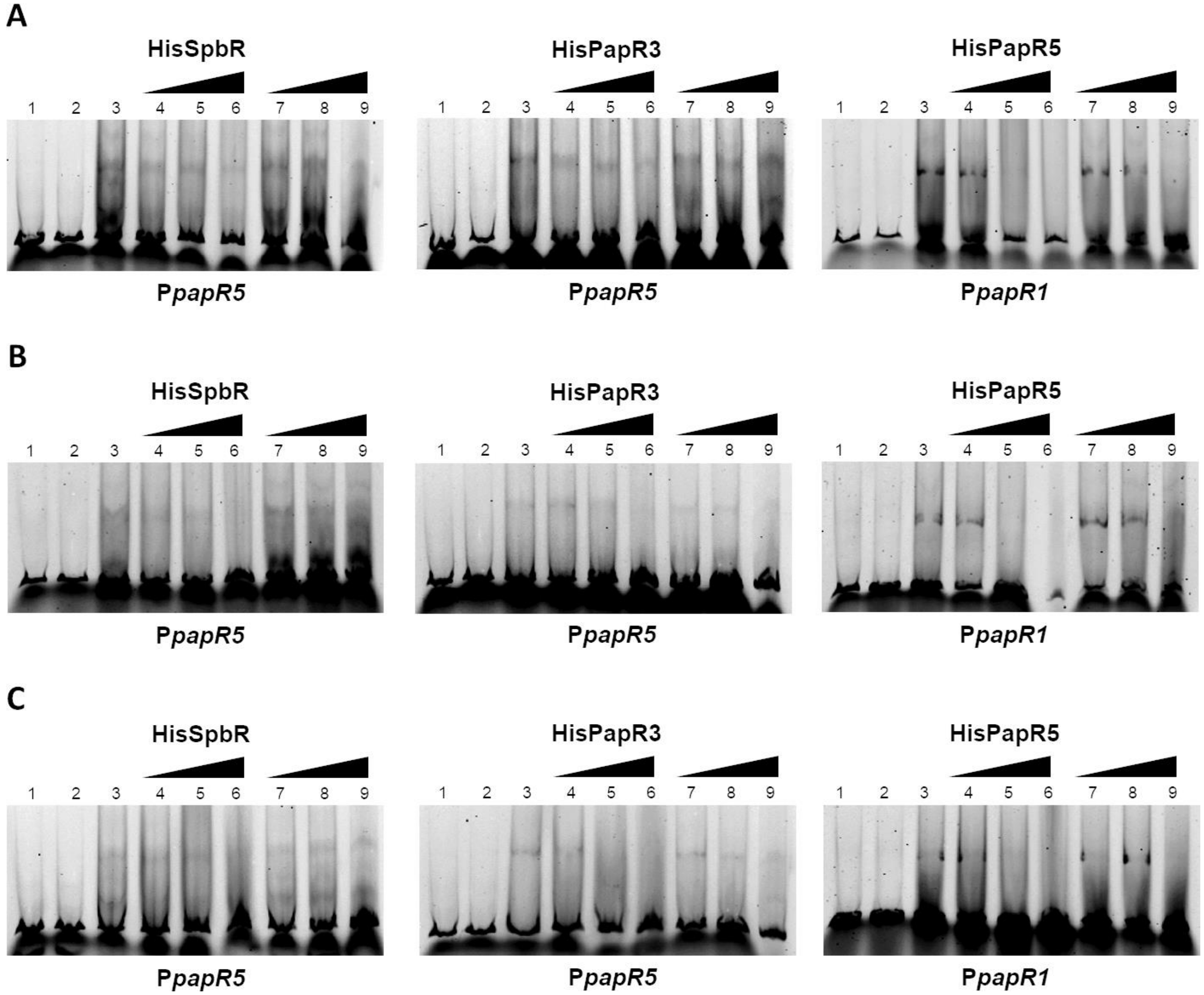

Supplementary Figure S2: EMSAs performed in 5% acrylamide gel with cell lysate samples from *SLspbR-OE* (left), *SLpapR3-OE* (middle) with P*papR5;* and *SLpapR5-OE* (right) with P*papR1* in the presence of *S. pristinaespiralis* WT culture extract (A: 30 h, B: 42 h, C: 48 h), respectively. 1 = promotor DNA, 2 = promotor DNA + *SLpGM190* lysate, 3 = promotor DNA + cell lysate from GBLR overexpression sample, 4-6 = addition of increasing concentration (1, 2, 4 µl) of *S. pristinaespiralis* WT culture extract, 7-9 = addition of increasing concentration (1, 2, 4 µl) of methanol as control.

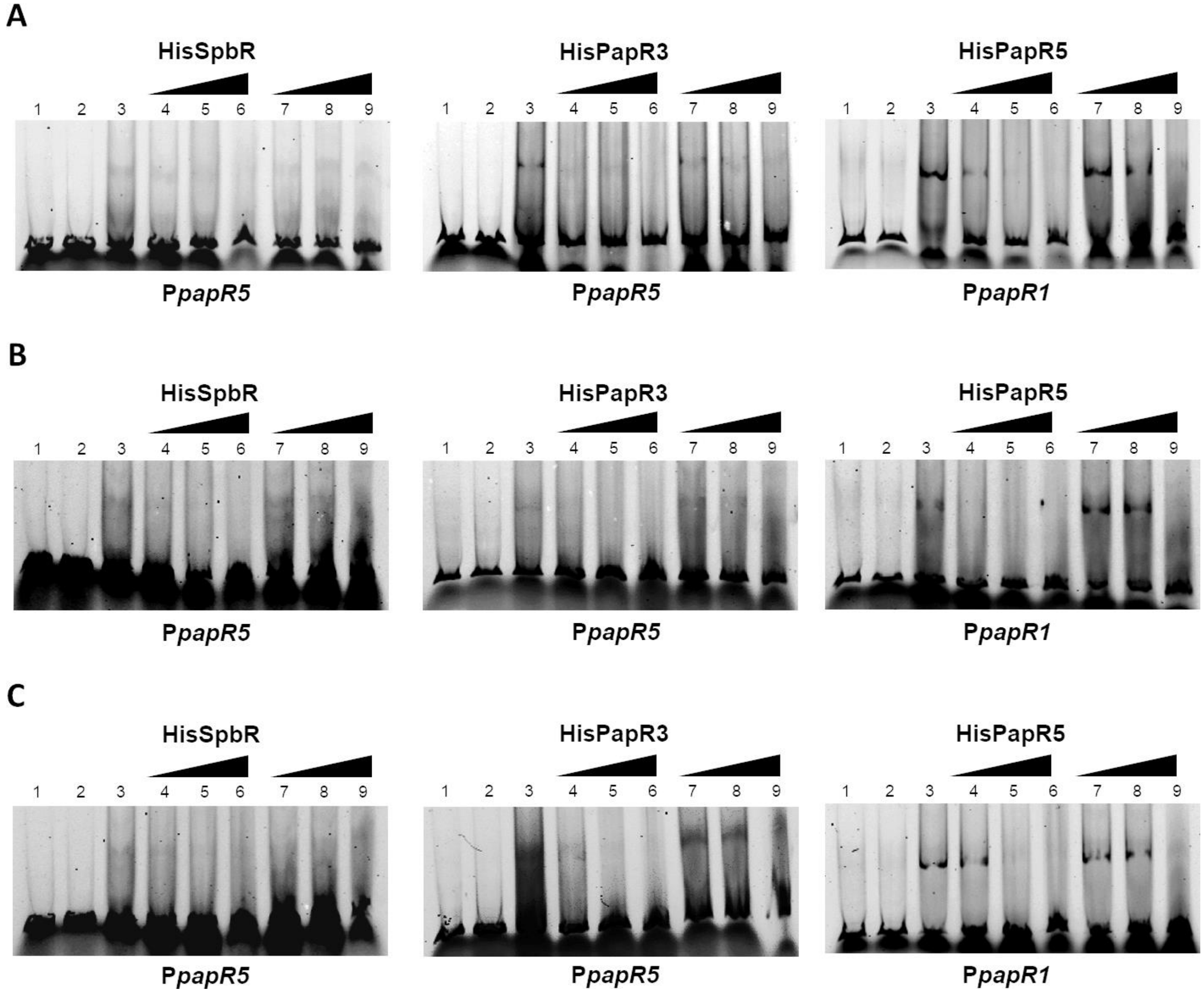

Supplementary Figure S3: EMSAs performed in 5% acrylamide gel with cell lysate samples from *SLspbR-OE* (left), *SLpapR3-OE* (middle) with P*papR5;* and *SLpapR5-OE* (right) with P*papR1* in the presence of *S. pristinaespiralis papR2::apra* culture extract (A: 30 h, B: 42 h, C: 48 h) , respectively. 1 = promotor DNA, 2 = promotor DNA + *SLpGM190* lysate, 3 = promotor DNA + cell lysate from GBLR overexpression sample, 4-6 = addition of increasing concentration (1, 2, 4 µl) of *S. pristinaespiralis papR2::apra* culture extract, 7-9 = addition of increasing concentration (1, 2, 4 µl) of methanol as control.

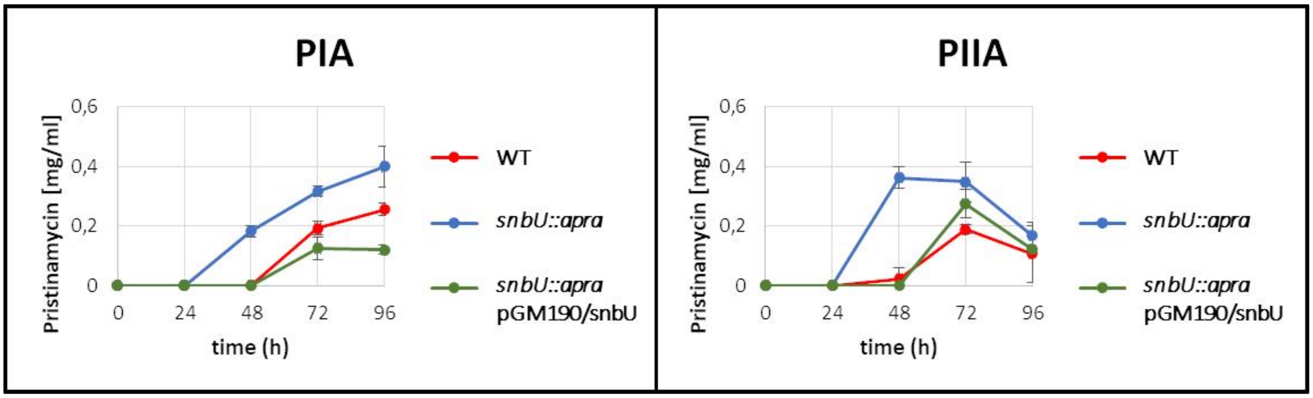

Supplementary Figure S4: Pristinamycin production (PIA (left), PIIA (right) of *S. pristinaespiralis* WT (red), the *snbU::apra* mutant (blue) and the complemented mutant *snbU::apra* with the expression construct pGM190/snbU (green) at different growth time points. Production curves are shown as the averages of three different experiments conducted in triplicate. Error bars indicate standard deviations.

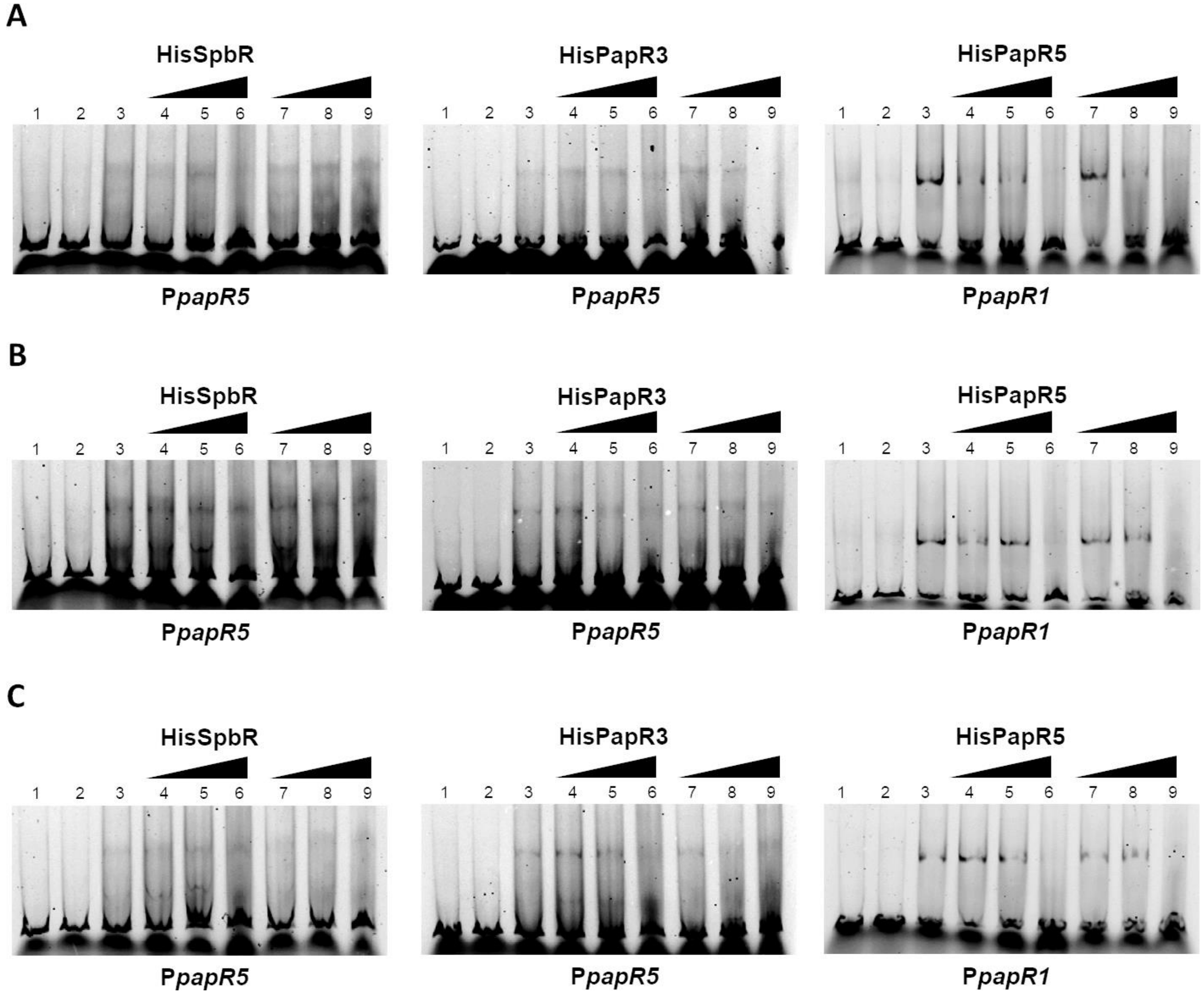

Supplementary Figure S5: EMSAs performed in 5% acrylamide gel with cell lysate samples from *SLspbR-OE* (left), *SLpapR3-OE* (middle) with P*papR5;* and *SLpapR5-OE* (right) with P*papR1* in the presence of *S. pristinaespiralis snbU::apra* culture extract (A: 30 h, B: 42 h, C: 48 h), respectively. 1 = promotor DNA, 2 = promotor DNA + *SLpGM190* lysate, 3 = promotor DNA + cell lysate from GBLR overexpression sample, 4-6 = addition of increasing concentration (1, 2, 4 µl) of *S. pristinaespiralis snbU::apra* culture extract, 7-9 = addition of increasing concentration (1, 2, 4 µl) of methanol as control.

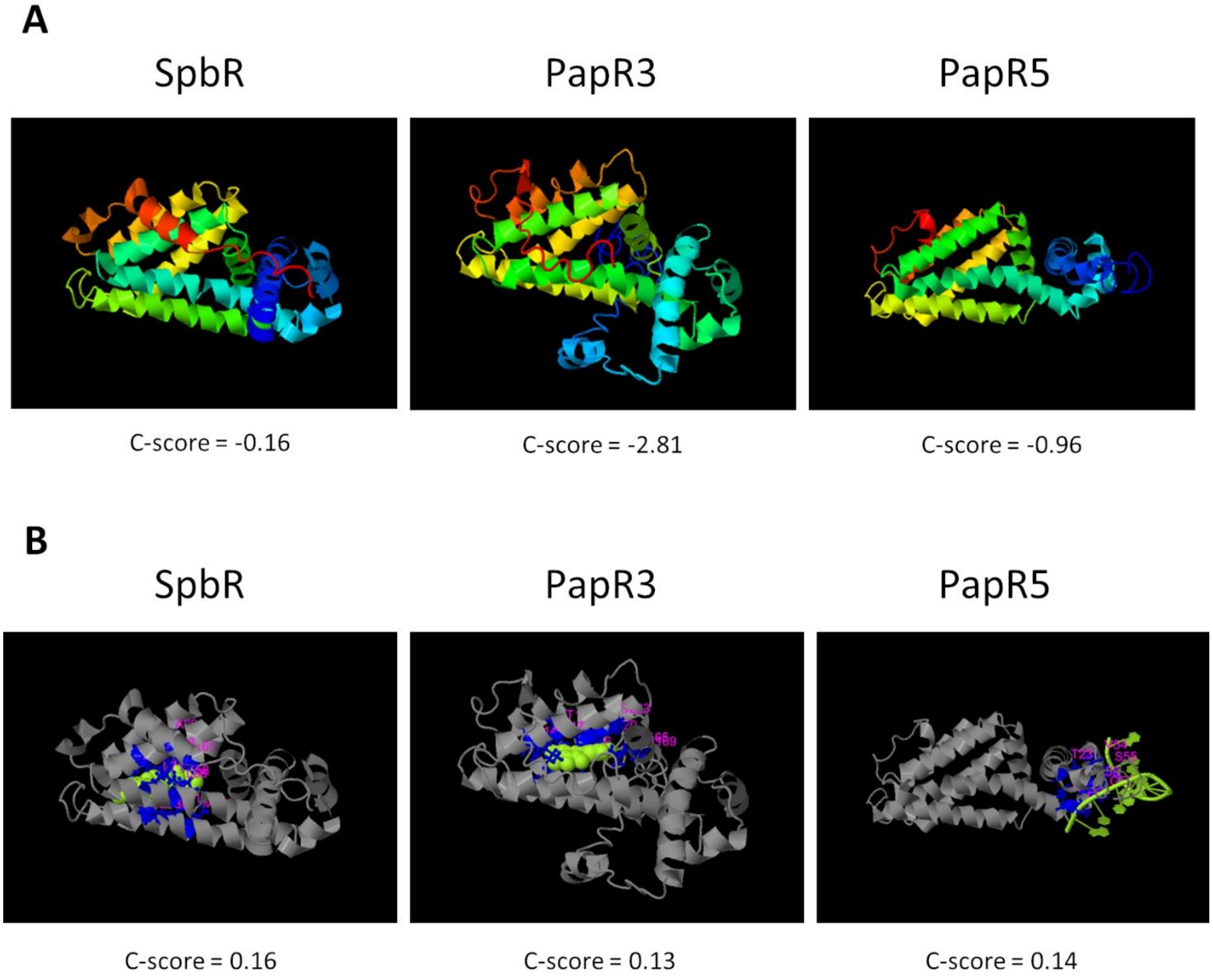

Supplementary Figure S6: 3D models for the GBLR-like proteins SpbR, PapR3, and PapR5 predicted by I-TASSER. The according confidence score (C-score) is given for estimating the quality of the predicted models. C-scores reside in the range of -5 and +1, where a C-score of higher value signifies a model with a high confidence (A). The predicted ligand-binding sites of the GBLR-like proteins SpbR, PapR3, and PapR5. The predicted binding ligands and ligand-binding residues are highlighted in yellow-green spheres and blue ball-and-sticks, respectively (B).

Supplementary Table S2: Potential ligands and ligand binding sites for the GBLR-like regulators SpbR, PapR3, and PapR5 predicted by I-TASSER.

SpbR

Rank	C-score	Ligand name	Ligand Binding Site Residues
1	0.16	3-[1-[4-(Methylaminomethyl)phenyl]piperidin-4-yl]-1-pyrrolidin-1-yl-propan-1-one	68,71,84,85,88,89,124,127,130,158,161,188
2	0.09	Nucleic Acids	10,41,42,44,45,48,49
3	0.08	Nucleic Acids	29,30,31,32,43,47,52,53
4	0.07	Erythromycin A	19,23,65,68,88,91,92,109,123,124,158,161,162
5	0.06	Phloretin	64,105,120,124,162

PapR3

Rank	C-score	Ligand name	Ligand Binding Site Residues
1	0.13	Quercetin	144,147,148,165,169,189,215,216,219,220,223
2	0.09	Nucleic Acids	68,99,100,101,102,103,106,107
3	0.09	Triclosan	123,126,148,168,169,181,182,185,189,215,216,219,223
4	0.06	Nucleic Acids	88,89,90,101,105,110,111
5	0.05	Triclosan	119,123,126,155,165,168

PapR5

Rank	C-score	Ligand name	Ligand Binding Site Residues
1	0.14	Nucleic Acids	23,54,55,57,58,61,62
2	0.10	Naringenin	77,106,120,142,168,169,172,176

3	0.09	Nucleic Acids	42,44,45,56,57,60,65,66
4	0.07	Phloretin	85,88,89,99,102,106,138,142,145,169,172,176
5	0.06	Erythromycin A	32,36,78,81,106,110,134,135,169,172,173

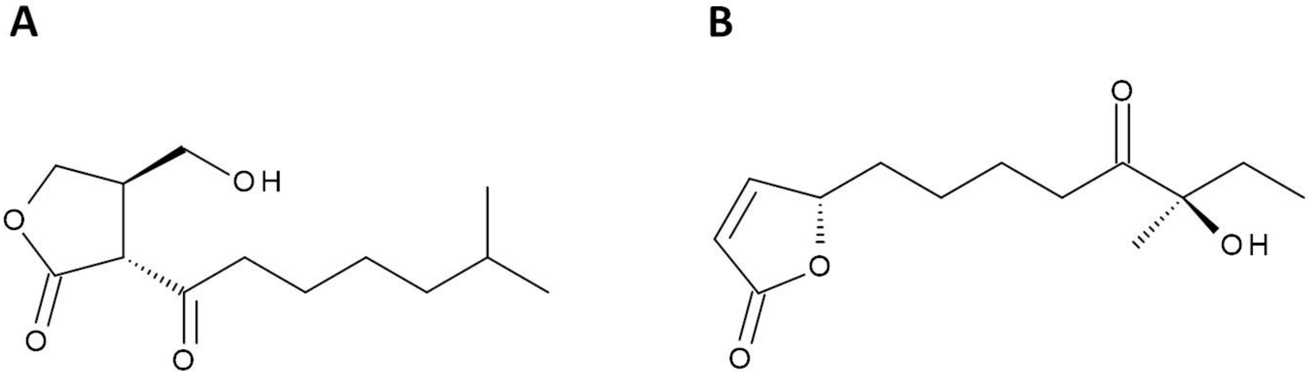

Supplementary Figure S7: Chemical structures of the A-factor from *Streptomyces coelicolor* (A) and avenolide from *Streptomyces avermitilis* (B).

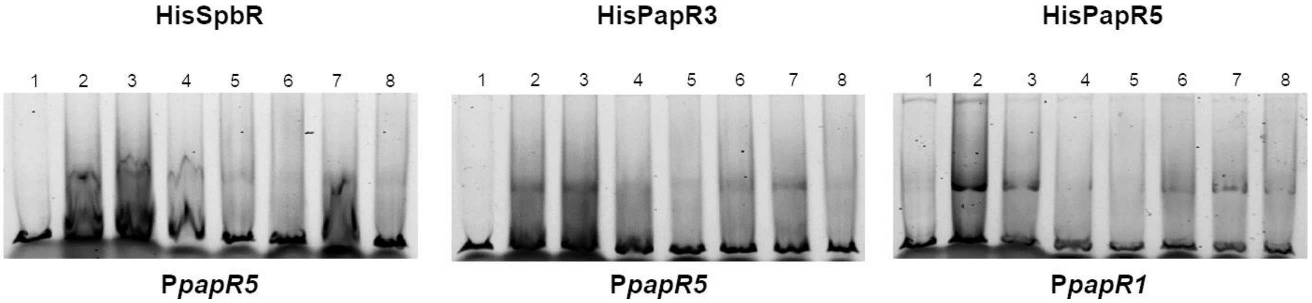

Supplementary Figure S8: EMSAs performed in 5% acrylamide gel under conditions as described in Du et al. (2011) with cell lysate samples from *SLspbR-OE* (left), *SLpapR3-OE* (middle) with P*papR5;* and *SLpapR5-OE* (right) with P*papR1* in the presence of *S. pristinaespiralis* WT culture extract, respectively. 1 = promotor DNA, 2 = promotor DNA + cell lysate from GBLR overexpression sample, 3 = addition of 2 µl of methanol as control, 4-8 = addition of 2 µl *S. pristinaespiralis* WT culture extract exposed to various treatments (5 = heat, 6 = acid, 7 = alkali, 8 = proteinase K).
